# Altered effective connectivity within an oculomotor control network in individuals with schizophrenia

**DOI:** 10.1016/j.nicl.2021.102764

**Published:** 2021-07-14

**Authors:** Matthew Lehet, Ivy F. Tso, Sebastiaan F.W. Neggers, Ilse A. Thompson, Beier Yao, René S. Kahn, Katharine N. Thakkar

**Affiliations:** a Department of Psychology, Michigan State University, East Lansing, MI, USA; b Department of Psychiatry, University of Michigan, Ann Arbor, MI, USA; c Department of Psychiatry, University Medical Center Utrecht, Utrecht, the Netherlands; d Department of Psychiatry, Icahn School of Medicine at Mount Sinai, New York, NY, USA; e Department of Psychiatry and Biobehavioral Medicine, Michigan State University, Grand Rapids, MI, USA

**Keywords:** SSRT, Stop-signal reaction time, TSRT, Target-step reaction time, TSD, Target-step delay, RT, Reaction time, FEF, Frontal eye fields, SC, Superior colliculi, SEF, Supplementary eye fields, IFC, Right inferior frontal cortex, DCM, Dynamic causal modeling, fMRI, functional magnetic resonance imaging, SZP, Persons with schizophrenia or schizoaffective disorder, HC, Healthy controls, PANSS, Positive and Negative Syndrome Scale, Schizophrenia, Dynamic causal modeling, Response inhibition, Eye movements, Effective connectivity, Stop-signal task, Executive function

## Abstract

Rapid inhibition or modification of actions is a crucial cognitive ability, which is impaired in persons with schizophrenia (SZP). Primate neurophysiology studies have identified a network of brain regions that subserves control over gaze. Here, we examine effective connectivity within this oculomotor control network in SZP and healthy controls (HC). During fMRI, participants performed a stop-signal task variant in which they were instructed to saccade to a visual target (no-step trials) unless a second target appeared (redirect trials); on redirect trials, participants were instructed to inhibit the planned saccade and redirect to the new target. We compared functional responses on redirect trials to no-step trials and used dynamic causal modelling (DCM) to examine group differences in network effective connectivity. Behaviorally, SZP were less efficient at inhibiting, which was related to their employment status. Compared to HC, they showed a smaller difference in activity between redirect trials and no-step trials in frontal eye fields (FEF), supplementary eye fields (SEF), inferior frontal cortex (IFC), thalamus, and caudate. DCM analyses revealed widespread group differences in effective connectivity across the task, including different patterns of self-inhibition in many nodes in SZP. Group differences in how effective connectivity was modulated on redirect trials revealed differences between the FEF and SEF, between the SEF and IFC, between the superior colliculus and the thalamus, and self-inhibition within the FEF and caudate. These results provide insight into the neural mechanisms of inefficient inhibitory control in individuals with schizophrenia.

## Highlights

• Rapid control of planned gaze shift impaired in individuals with schizophrenia (SZ).
• Current study aimed to investigate neural basis of these impairments.
• SZ less efficient at inhibiting gaze shift, which was related to employment.
• SZ showed altered activation in oculomotor control network during task performance.
• Effective connectivity and its modulation by inhibitory demands altered in SZ.

## 1 Introduction

Executive control is a crucial cognitive ability that permits flexible responses to changing environmental demands. Individuals with schizophrenia show deficits in executive control, which are, importantly, related to poor social outcomes (Barch and Sheffield, 2017, Bilder et al., 2000, Green et al., 2000, Nuechterlein et al., 2004, Shin et al., 2013). Accordingly, executive control deficits are targets for behavioral, pharmacological, and neurostimulation interventions (Minzenberg & Carter, 2012), and these treatment efforts would benefit from a deeper understanding of both the nature and neural mechanisms of executive control impairments in the illness. The stop-signal task and related paradigms measure one important component of executive control: the ability to reactively inhibit or modify a planned action. These paradigms provide ideal measures of executive control for two major reasons: 1) task behavior can be described using formal mathematical models, thus allowing for a potentially more nuanced glimpse into executive control abnormalities; and 2) this task has been used across species and thus provides a translational bridge for understanding the neural mechanisms of executive control deficits in clinical populations.

The stop-signal task requires participants to make a speeded response to a signal (go trial). On some trials, however, a second signal is presented shortly after the first, which instructs the participant to stop or change the prepared response (stop trial). Performance on this task can be modeled as a race between competing GO and STOP processes, which permits calculation of the time it takes for the STOP process to complete (i.e., the speed of inhibition)—the stop-signal reaction time (SSRT; Logan & Cowan, 1984). Longer SSRT has been reported in individuals with schizophrenia measured by both keypress (Huddy et al., 2009, Hughes et al., 2012, Nolan et al., 2011, Shin et al., 2013) and eye movement responses (Thakkar et al., 2011, Thakkar et al., 2015a, Thakkar et al., 2015b) and has been related to both symptom severity (Hutton et al., 2004, Shin et al., 2013, Thakkar et al., 2011, Thakkar et al., 2015b, Van Voorhis et al., 2019) and occupational functioning (Thakkar et al., 2011, Thakkar et al., 2015b). An advantage of using eye movement responses is the rich body of non-human primate neurophysiology work that has characterized the role of single neurons in reactively stopping or changing a planned eye movement under similar conditions to those used in studies with human participants, which can be leveraged to better understand findings in clinical populations.

In non-human primates performing the stop-signal task, activity in movement neurons in the frontal eye fields (FEFs) and superior colliculi (SC) quickly attenuates following presentation of the stop-signal (Brown et al., 2008, Hanes et al., 1998, Paré and Hanes, 2003), whereas activity in fixation neurons (which are active while the monkey is fixating) increases (Hanes and Schall, 1995, Xu et al., 2017). Modulation of movement activity might occur via interactions between movement and fixation neurons within the FEF and SC. Alternatively, basal ganglia output can inhibit the SC directly and the FEF via the thalamus, effectively inhibiting saccades (Hikosaka et al., 2000, Schmidt et al., 2013, Watanabe and Munoz, 2010).

The role of the medial frontal cortex in reactive control of actions has been more controversial. Medial frontal cortex encompasses the supplementary motor complex, which includes the supplementary and pre-supplementary motor areas and the supplementary eye fields (SEF). Non-human primate work has revealed modulation of firing in the SEF following presentation of the stop signal, but the pattern of findings suggests that this modulation reflects a later evaluative process (Pouget et al., 2017, Stuphorn et al., 2010, Stuphorn and Emeric, 2012) rather than a direct inhibitory process. Such evaluation includes assessing the consequence of actions by representing expected and actual reward (Pouget et al., 2017, So and Stuphorn, 2012), the implementation of risk aversion preferences (Chen & Stuphorn, 2018), and decision confidence (So and Stuphorn, 2012, So and Stuphorn, 2016). Based on these assessments, the SEF regulates saccade likelihood by changing the balance between fixation and saccade execution (Stuphorn et al., 2010, Stuphorn and Schall, 2006, Stuphorn et al., 2000). On the basis of functional MRI studies, however, the medial frontal cortex—and the supplementary motor complex in particular-- has been argued to play a more direct role in reactive inhibition in humans, possibly through connections to primary motor regions via the basal ganglia (Duann et al., 2009, Zandbelt et al., 2013).

Finally, the right inferior frontal cortex (IFC) has been implicated in early stages of inhibitory processes (Aron et al., 2014, Cai et al., 2014, Cieslik et al., 2015, Wessel and Aron, 2017, Zhang et al., 2017), including the inhibition of eye movements (Thakkar et al., 2014, Xu et al., 2017). In coordination with the supplementary motor complex and anterior cingulate, the IFC is positioned to coordinate stopping processes through projections to the sub-thalamic nucleus (Aron et al., 2016, Jahfari et al., 2011, Mallet et al., 2016, Utter and Basso, 2008, Wiecki and Frank, 2013). Despite this proposed mechanism, whether the right IFC response indeed instantiates outright stopping or represents the detection of the salient stop signal remains controversial (Hampshire and Sharp, 2015, Sebastian et al., 2018).

Thus, these aforementioned brain regions (FEF, SEF, right IFC, caudate, thalamus, and SC) comprise a putative circuit involved in the reactive control of saccadic eye movements—a claim substantiated by anatomical connections between these regions. FEF and SEF have reciprocal connections (Huerta and Kaas, 1990, Huerta et al., 1987, Parthasarathy et al., 1992), and both have descending connections to SC (Collins et al., 2005, Meredith, 1999, Shook et al., 1990), striatum (Cui et al., 2003, Griggs et al., 2017, Huerta and Kaas, 1990, Parthasarathy et al., 1992, Shook et al., 1991), and thalamus (Huerta and Kaas, 1990, Orem and Schlag, 1971, Parthasarathy et al., 1992, Shook et al., 1991). Ascending connections from the striatum and the SC to the FEF and SEF are routed through the thalamus (Lynch et al., 1994, May, 2006, Tanaka and Kunimatsu, 2011). SC and basal ganglia are reciprocally connected (Comoli et al., 2003, Hikosaka et al., 2000, McHaffie et al., 2006, Redgrave et al., 2010), with additional connections from the thalamus to the SC (Rieck et al., 1986). Although not a traditional oculomotor region, the IFC projects to the caudate (Griggs et al., 2017), and has bidirectional connections to the SEF (Huerta and Kaas, 1990, Petrides and Pandya, 2002) and thalamus (Kievit & Kuypers, 1977), and in this way may implement control over saccades.

Collectively, this work provides a robust framework to understand deficits in executive control over response execution in individuals with schizophrenia. More specifically, understanding the dynamic interactions within a circuit involved in the rapid control of eye movements—substantiated by both human and animal work—may provide new insights into the mechanisms of executive control impairments in schizophrenia. To date, however, studies examining the neural correlates of stop-signal task impairments in schizophrenia have primarily focused on modular, segmented explanations within discrete regions. Several EEG studies have revealed reduced amplitude of sensory and motor preparatory responses to the signal to go and stop (Hoptman et al., 2018, Hughes et al., 2012, Van Voorhis et al., 2019) and later evaluative responses (Hoptman et al., 2018, Hughes et al., 2012, Van Voorhis et al., 2019, Yu et al., 2019). Functional MRI and functional near infrared spectroscopy studies paint a less clear picture, partly because studies vary in the tasks employed and the contrasts examined. functional near infrared spectroscopy studies have shown that individuals with schizophrenia show reduced recruitment of ventrolateral prefrontal cortex, including IFC, during stop-signal task performance (Okada et al., 2016, Tsujii et al., 2018). Findings from fMRI studies that have compared successful stop trials to go trials have yielded mixed results, with some studies finding reduced differential activation of the IFC (Hughes et al., 2012) and medial frontal cortex (Rubia et al., 2001) in individuals with schizophrenia compared to controls, others finding greater activation in IFC and medial prefrontal cortex in patients (Lindberg et al., 2016), and others reporting no difference (Moran et al., 2018, Zandbelt et al., 2011). These findings point to altered patterns of brain activity during stop-signal task performance in individuals with schizophrenia; however, results are mixed.

Examining connectivity between regions may help elucidate the mixed neuroimaging results. Functional connectivity studies suggest aberrant connectivity between striatal, frontal-parietal, and sensorimotor networks in individuals with schizophrenia performing the stop-signal task, which related to performance (Hoptman et al., 2018, Moran et al., 2018, Yang et al., 2020). Although these studies reveal altered correlations in activity between different brain regions, they do not permit inferences about causal interactions, or effective connectivity. Dynamic causal modeling (DCM) is a methodological approach that aims to do exactly that: estimate the coupling among brain regions and how that coupling is modulated by experimental context (Friston, 2020, Friston et al., 2016, Zeidman et al., 2019a, Zeidman et al., 2019b). Understanding differences between individuals with schizophrenia and healthy controls in the causal architecture of a putative oculomotor control network during the reactive inhibition of saccades may provide deeper insights into the mechanisms of executive control dysfunction.

In the present study, we build upon existing work in several important ways that we believe will advance the understanding of mechanisms of reactive inhibition impairments in individuals with schizophrenia. First, the bulk of the reviewed neuroimaging stop-signal studies in individuals with schizophrenia use keypress responses. In the current study, we employ a stop-signal task variant that requires control over eye movements during fMRI, thus rendering our results more directly comparable with work in non-human primates. Second, we employ dynamic causal modeling to measure *causal* interactions (i.e., effective connectivity) between regions that subserve behavioral inhibition. We use findings from single-cell recordings in non-human primates performing the stop-signal task, human fMRI studies of stop-signal task performance, and anatomical tracing studies in animals to motivate our models. More specifically, we investigated activation in an oculomotor control network (i.e., FEF, SEF, IFC, SC, thalamus, and the caudate nucleus of the basal ganglia) as well as effective connectivity between nodes in this network in individuals with schizophrenia or schizoaffective disorder and healthy controls performing a modified oculomotor stop-signal task. In this so-called search-step task, a target is presented amongst an array of distractors and the participant is instructed to look at the target as quickly as possible. On a minority of trials, the target jumps to a new location and the participant is instructed to inhibit the saccade to the initial target location and instead redirect gaze to the new target location. Using race model logic, the latency of inhibition can be quantified and it is referred to as the target step reaction time (TSRT; Camalier et al., 2007, Murthy et al., 2007, Thakkar et al., 2014). Like SSRT, individuals with schizophrenia have been shown to have longer TSRT (Thakkar et al., 2015b). On the basis of prior work, we developed the following hypotheses. First, we expected to replicate our previous behavioral findings describing longer TSRT/SSRT in individuals with schizophrenia and relationships between longer TSRT/SSRT and unemployment in patients (Thakkar et al., 2011, Thakkar et al., 2015b). We also expect to see reduced activation for patients in this specified oculomotor control network on trials where participants are instructed to redirect a planned saccade versus simply executing a visually-guided saccade. Finally, we expected that the instruction to redirect a saccade would modulate causal connections in this oculomotor network differently in individuals with schizophrenia as compared to healthy controls, particularly in fronto-striatal-thalamic circuits where dysfunction has been widely reported (Quidé et al., 2013, Sarpal et al., 2015, Segarra et al., 2016, Zandbelt et al., 2011, Öngür et al., 2010). Better characterizing the nature of and neural mechanisms underlying inhibitory control deficits commonly seen in individuals with schizophrenia may provide translational insights from animal models – where saccadic control tasks are well-characterized – into clinical treatments.

## 2 Material and methods

### 2.1 Participants

Twenty-one antipsychotic-medicated persons with schizophrenia or schizoaffective disorder (SZP) were recruited from a longitudinal study (Genetic Risk and Outcome in Psychosis (GROUP) Investigators, 2011) and an outpatient psychiatric facility in The Netherlands. Twenty-four demographically-matched healthy controls (HC) without a personal or family history of an Axis I psychiatric diagnosis (Diagnostic and Statistical Manual of Mental Disorders (DSM-IV), fourth edition) were selected based on age and gender from a larger group of HC participants recruited via community advertisements. All participants were screened to exclude a history of head trauma or neurological illness, recent substance abuse or dependence, and color blindness. SZP and HC were matched for age, sex, IQ, and handedness. See Table 1 for demographic information. All subjects gave written informed consent and were reimbursed for participation. The study was approved by the Human Ethics Committee of the University Medical Center, Utrecht.

**Table 1:** Demographic Information.

	HC (n = 24) mean (s.d.)	SZP (n = 21) mean (s.d.)	statistic	p-value
**Age**	33.9 (8.5)	37.0 (8.0)	t = 1.25	0.22
**Sex**	15 M / 9F	15 M / 6F	χ2 = 0.40	0.53
**IQ**^1^	100.0 (14.5)	96.4 (11.9)	t = 0.84	0.4
**Handedness**^2^	0.72 (0.63)	0.93 (0.19)	t = 1.50	0.14
**Education**^3^	6.92 (1.56)	4.81 (1.72)	t = 4.31	<0.0001
**Illness duration (years)**		14.40 (5.12)		
**CPZ Equivalent (mg)**		278.50 (249.88)		
**PANSS Positive**		12.10 (5.10)		
**PANSS Negative**		13.05 (6.41)		
**PANSS General**		25.81 (7.79)		
**PANSS Total**		51.05 (17.24)		
**SFS total**		788.36 (59.00)		
**SFS employment**		108.10 (10.21)		

CPZ, chlorpromazine; HC, healthy control subjects; PANSS, Positive and Negative Syndrome Scale; SFS, Social Functioning Scale; SZP, persons with schizophrenia

1 Based on the Nederlandse Leestest voor Volwassenen

2 Based on the Edinburgh Handedness Inventory; scores range from 0 indicating complete left-handedness to 1 indicating complete right-handedness.

3 Education category: 0 = <6 years of primary education; 1 = finished 6 years of primary education; 2 = 6 years of primary education and low-level secondary education; 3 = 4 years of low-level secondary education; 4 = 4 years of average-level secondary education; 5 = 5 years of average-level secondary education; 6 = 4 years of secondary vocational training; 7 = 4 years of high-level professional education; 8 = university degree.

### 2.2 Assessment

Schizophrenia or schizoaffective disorder diagnoses were based on DSM-IV criteria and verified with the Comprehensive Assessment of Symptoms and History interview (Andreasen et al., 1992) or Schedules for Clinical Assessment for Neuropsychiatry, version 2.1 (Wing, 1990). Chlorpromazine (CPZ) equivalent antipsychotic dosages were calculated for each patient (Woods, 2003). Clinical symptoms in patients were assessed with the Positive and Negative Syndrome Scale (PANSS; Kay et al., 1987). PANSS total scores corresponded to a Clinical Global Impressions (CGI) severity rating of ‘Borderline Mentally Ill’ (Leucht et al., 2005). Positive, negative, and general symptom subscores were calculated. Premorbid IQ was assessed with a word reading test, the Nederlandse Leestest voor Volwassenen (NLV; Schmand et al., 1991). Social and occupational functioning was assessed in patients using the Dutch translation of the Social Functioning Scale (SFS; Birchwood et al., 1990). Raw scores were standardized (mean = 100, s.d. = 15) based on normative data from patients with schizophrenia (e.g., Birchwood et al., 1990). We were particularly interested in the occupational activity subscale given its previous relation to inhibition speed (Thakkar et al., 2011, Thakkar et al., 2015a, Thakkar et al., 2015b).

### 2.3 Saccadic search-step task

**Fig. 1:**
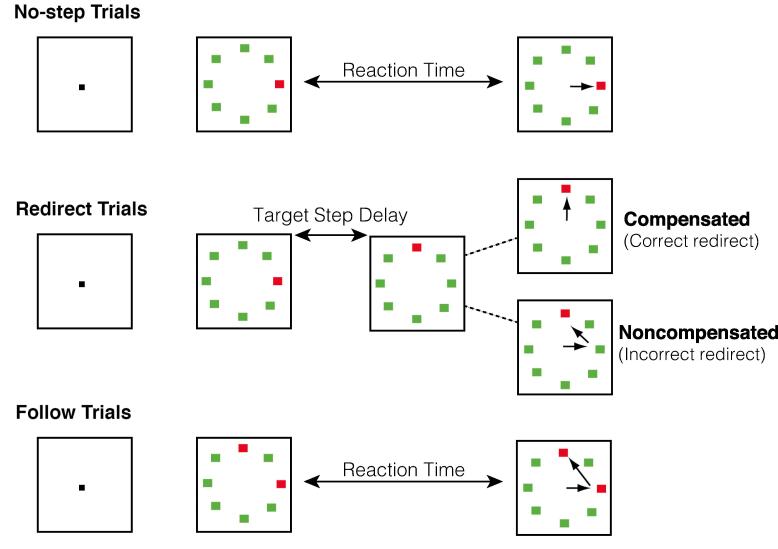
Search-step task. The task consisted of three randomly interleaved trial types: *no-step* (30% of trials), *redirect* (40% of trials), and *follow* (30% of trials). Each trial lasted 4 s and began with a variable fixation period between 1000 and 2000 ms. On no-step and redirect trials, after the fixation period, an eight-element search array appeared with one red singleton among green distractors (T1). On no-step trials, this array remained on the screen for the remainder of the trial. On redirect trials, the red target jumped to a new location (T2) via an isoluminant color change at some delay after the initial array presentation (target step delay; TSD). On follow trials, the array appeared with two red targets and was visible until the end of the trial. On no-step and redirect trials, subjects were instructed to saccade to T1 as quickly as possible. They were instructed that if the target jumped to a new location (redirect trials), they should try to inhibit the saccade to T1 and to look as quickly as possible to T2. On follow trials, participants were instructed to look at each red target in succession (the order was irrelevant). Redirect trials in which the subject successfully looked immediately toward T2 were referred to as *compensated trials*. Redirect trials in which the subject erroneously made an initial saccade to T1 were referred to as *noncompensated trials*; these noncompensated trials were nearly always followed by a corrective saccade to the new target location. (For interpretation of the references to color in this figure legend, the reader is referred to the web version of this article.)

Participants performed a saccadic search-step task (Fig. 1; Camalier et al., 2007, Murthy et al., 2007) that consisted of three randomly interleaved trial types: *no-step* (30% of trials), *redirect* (40% of trials), and *follow* (30% of trials). Each trial lasted 4 s and began with a variable fixation period between 1000 and 2000 ms. On no-step and redirect trials, after the fixation period, an eight-element search array appeared with one red singleton among green distractors. The array elements subtended 0.7° of visual angle and were isoluminant and equidistant from the center (9° of visual angle). On no-step trials, this array remained on the screen for the remainder of the trial. On redirect trials, the red target jumped to a new location via an isoluminant color change at some delay after the initial array presentation (target step delay; TSD). On follow trials, the array appeared with two red targets and was visible until the end of the trial. On no-step and redirect trials, subjects were instructed to saccade to the red target (T1) as quickly as possible. They were instructed that if the target jumped to a new location (redirect trials), they should try to inhibit the saccade to T1 and to look as quickly as possible to the new target location (T2). On follow trials, participants were instructed to look at each red target in succession (the order was irrelevant). Follow trials were included for analyses of error-related activity, which is not directly relevant to the current research questions; thus, activity during these trials is not discussed. Redirect trials in which the subject successfully looked immediately toward T2 were referred to as *compensated trials*. Redirect trials in which the subject erroneously made an initial saccade to T1 were referred to as *noncompensated trials*. Inhibition of the saccade to T1 becomes more difficult with increasing TSDs (Camalier et al., 2007, Logan and Cowan, 1984). The TSDs were dynamically adjusted with a one-up/one-down tracking procedure, thereby ensuring successful inhibition on approximately 50% of the redirect trials. The initial TSD was set at 100 ms and increased or decreased by 67 ms when the subject succeeded or failed to inhibit, respectively. TSDs were multiples of the screen refresh rate to minimize timing inaccuracy. To minimize the occurrence of averaging saccades landing midway between T1 and T2, target locations were constrained on redirect and follow trials such that there was at least 90° between T1 and T2 (Van der Stigchel & Nijboer, 2011).

Trials were presented in four 5-min experimental sessions consisting of 60 trials each. In each session, six 10 s rest blocks displaying only the fixation cross were interleaved as a null condition. Simulations were run before the experiment to determine a trial order in which correlations between the different model regressors was sufficiently low to allow for reliable estimation of parameters. In total, 72 no-step trials, 72 follow trials, and 96 redirect trials were presented. Participants were trained on the search-step task outside of the scanner prior to the fMRI experiment. In order to minimize the strategy of waiting for the target to move to a new location, we instructed participants that speed on the no-step and follow trials was equally as important as successfully inhibiting a saccade to T1 on redirect trials and that it would not always be possible to inhibit the saccade to T1 on redirect trials. Participants were not explicitly instructed about the relative frequency of trial types.

### 2.4 Stimulus display and eye tracking

Stimuli were displayed using Presentation software (Neurobehavioral Systems) and presented on an MR-compatible LED screen at the rear of the bore that was viewed by the participant via a mirror on the head coil. Eye movements were recorded during scanning using an MR-compatible infrared camera (Nordic Neuro Lab). This system used a video camera mounted to the head coil, with the infrared illumination being provided by LEDs that were also mounted on the head coil. Eye position was sampled at a rate of 60 Hz. Acquisition was controlled by ViewPoint eye-tracking software (Arrington Research). Stimuli presented by Presentation were digitally encoded and relayed to the ViewPoint software as triggers that were inserted into the eye movement recordings. Eye position data from each trial were stored and analyzed online to determine accuracy of redirect trials and adaptively adjust the TSD. After each redirect trial, eye position data were drift-corrected using the mean eye position in a window from 50 ms before and after array presentation. A positional criterion was used to determine trial accuracy. If the eye position moved outside of a window spanning 2° of visual angle around fixation after 100 ms for at least two samples (33 ms) and was in the direction of T2, then the trial was classified as compensated and the TSD was increased on the following redirect trial. If the eye position was in the direction of T1, the trial was classified as noncompensated and the TSD was decreased on the next redirect trial. If the eye position was not in the direction of either T1 or T2 (perhaps due to a blink or noise in the eye trace), the TSD remained the same.

### 2.5 Eye tracking data analysis

#### 2.5.1 Eye position data

Eye position data were analyzed offline using a semi-automated MATLAB procedure (The MathWorks). First, eye position data were differentiated to obtain a velocity signal and then filtered with a fifth-order Butterworth filter (40 Hz cutoff). Then, saccade onsets were determined automatically using liberal velocity criteria. After this automated procedure, erroneously marked saccades (e.g., camera noise, head movements, blinks, etc.) were removed manually. Verification of saccade onsets was performed blind to the experimental condition. Trials in which saccades were initiated < 100 ms after array onset were excluded from further analysis. Directional accuracy of saccades relative to the required response was determined using an automated procedure. Saccade latency on no-step and noncompensated trials was calculated as the onset of the saccade relative to array onset. Latency of compensated trial saccades was calculated as the onset of the saccade relative to T2 onset.

#### 2.5.2 Task performance

Behavioral performance was evaluated through measurements of saccadic RT on no-step, compensated, and noncompensated trials and TSD. At each TSD, the proportion of trials in which a participant correctly made a saccade immediately to T2 was quantified. Performance in the search-step task can be accounted for by a mathematical model that assumes a race between independent processes that generate (GO1) and inhibit (STOP) the movement to the initial target location (Camalier et al., 2007, Logan and Cowan, 1984). The response to T1 is executed if the GO1 process finishes first and inhibited if the STOP process finishes first. The latency of the GO1 process can be measured directly from the observable reaction times (RTs) to look at T1, but the latency of the STOP process must be estimated. The independent race model provides an estimate of the time needed to respond to the target step and cancel the saccade to T1 (i.e., the time needed for the STOP process to complete), which is referred to as the target step reaction time (TSRT). It is an analogous measure to stop-signal reaction time (SSRT) in the standard stop-signal paradigm (Hanes and Schall, 1995, Thakkar et al., 2011, Thakkar et al., 2015a). TSRT was calculated using the integration method (Congdon et al., 2012, Logan and Cowan, 1984, Verbruggen et al., 2013), which is the least biased and most reliable method for estimating TSRT/SSRT when combined with a dynamic tracking procedure (Verbruggen et al., 2019). To compute TSRT using this procedure, RTs on no-step trials were sorted in ascending order and the RT corresponding to the proportion of noncompensated trials was selected. The mean TSD was then subtracted from this RT.

#### 2.5.3 Statistical analyses

Given previous findings of longer inhibition latency in schizophrenia (Enticott et al., 2008, Huddy et al., 2009, Thakkar et al., 2011, Thakkar et al., 2015a, Thakkar et al., 2015b), one-tailed independent t-tests were used to compare TSRT between groups. All other tests were two-tailed. The proportion of compensated trials was compared across groups using an independent *t*-test. RT was examined using a mixed-model ANOVA, including trial (no-step, compensated, noncompensated) as a within-subjects variable and diagnostic group as a between-subjects variable. Greenhouse-Geisser adjustments of degrees of freedom were performed to correct for sphericity violations. Relationships between task performance and occupational functioning (SFS employment subscale) were investigated in SZP only using Spearman’s correlation coefficients.

### 2.6 fMRI data acquisition and analysis

#### 2.6.1 Data acquisition

The experiment was performed on a 3.0 T Achieva MRI scanner (Philips Medical Systems) at the University Medical Center Utrecht. Images were acquired using an eight-channel sensitivity-encoding (SENSE) parallel imaging head coil. Whole-brain T2*-weighted echo planar images with blood-oxygen level-dependent (BOLD) contrast (4 sessions; 152 volumes; 35 slices per volume; interleaved acquisition; TR 2 s; TE 35 ms; field of view 256 × 256 × 120 mm; flip angle 70°;96 96 35 matrix; voxel size 2.67 2.67 3.42; SENSE factor, 2.4 anterior–posterior) oriented in a transverse plane were acquired. The first six images were discarded to allow for T1 equilibration effects. A whole-brain three-dimensional fast-field echo T1-weighted scan (200 slices; TR 10 ms; TE 4.6 ms; flip angle 8°; field of view, 240 240 160 mm; voxel size: 0.75 0.8 0.75 mm) was acquired for within-subject registration purposes.

To remove cardiac and respiratory pulsality effects that contaminate BOLD fMRI time series, cardiac signals and respiration were measured using equipment built into the Philips Achieva scanner. Cardiac signals were measured at 500 Hz with ECG electrodes and respiration was recorded at 500 Hz using a band wrapped around participants’ midsection.

#### 2.6.2 Preprocessing

Functional imaging data were preprocessed and analyzed using SPM12 (http://www.fil.ion.ucl.ac.uk/spm/software/), AFNI (https://afni.nimh.nih.gov/), and MATLAB. First, the raw fMRI data were preprocessed spatially. Images were realigned to correct for head motion in the scanner using rigid body transformations and a mean functional image was created. Next, the data were temporally interpolated per slice to correct for the individual timing differences in slice acquisition such that the signal of each slice was interpolated to the time of acquisition of the middle slice. The anatomical image was co-registered to the mean functional image using the normalized mutual information criteria method. Segmentation and normalization of the anatomical image into Montreal Neurological Institute (MNI) space was achieved using a unified segmentation method (Ashburner & Friston, 2005). The same normalization parameters were applied to the functional scans, which were in register with the anatomical images. The fMRI images were spatially smoothed with a Gaussian kernel with a full width at half maximum (FWHM) of 6 mm. Finally, in order to remove remaining motion-related noise, the volumes were despiked using AFNI’s 3Ddespike function.

#### 2.6.3 Statistical analyses: first level general linear models

Statistical analysis was performed within the framework of the general linear model (GLM) and followed a two-level procedure. First-level statistical analysis involved modeling of no-step, follow, compensated, and noncompensated trials. Six 10 s Rest (fixation only) trials were also included in the design but were not explicitly modeled and therefore constituted an implicit baseline. Regressors were created by convolving delta functions coding the array onset with a canonical hemodynamic response function. Twenty nuisance regressors were added to model cardiac and respiratory pulsality using the RETROICOR method with fifth-order Fourier expansions (Glover et al., 2000). Physiological non-neuronal rhythms are known to have a robust effect on the BOLD signal, especially in midbrain and basal ganglia areas, due to, among other things, the arterial circle of Willis vasculature. Modeling such rhythms as covariates using RETROICOR increases sensitivity to neuronal activation of interest. Temporal autocorrelation in the fMRI data was modeled using autoregressive modeling of the first order by prewhitening the GLM equation. Data were also high-pass filtered during prewhitening with a cutoff cycle length of 70 s.

In determining our contrasts of interest, we were interested in how the processes associated with inhibiting and redirecting a planned saccade would change neural processing as compared to making a simple visually-guided saccade. Therefore, we focused our analysis on three contrasts: redirect versus fixation, no-step versus fixation, and redirect versus no-step. Our decision to collapse compensated and noncompensated trials was based on our study goal to understand putative differences in inhibitory processes, which would presumably transpire on both correctly compensated and incorrectly noncompensated trials. That is, according to race model logic, participants could fail to compensate on redirect trials because an initiated STOP process simply failed to “win” the race. This decision to collapse across trials is justified based on previous work from our lab (Thakkar et al., 2014) and others (Curtis et al., 2005) that reported no regions in which activity was greater on compensated trials, and also based on results from our current sample that also did not reveal greater activity on compensated trials than noncompensated trials in any of our a priori ROIs (see Supplementary Material).

#### 2.6.4 Statistical analyses: second level general linear models

First-level contrast images were analyzed in a whole-brain second-level random-effects analysis using one-sample *t* tests. These second-level contrast images were computed for HC, SZP, and the combined sample. These contrasts were examined in an exploratory whole brain analysis described in the Supplementary Material and results are also presented in *Table S1* and Figure S1.

**Fig. 2:**
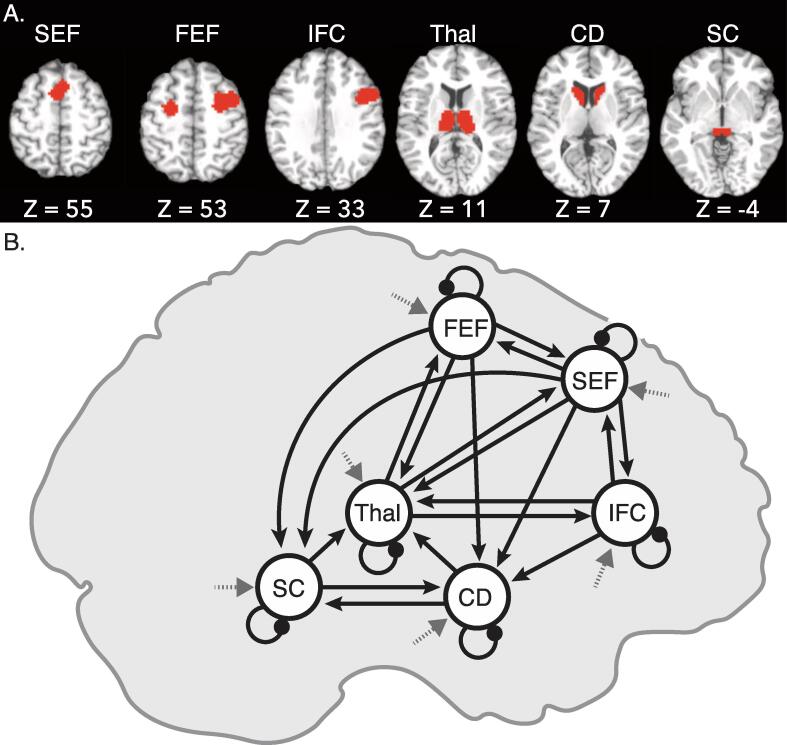
Regions of interest (ROIs) and the full model for dynamic causal modeling analysis (DCM). (A) ROI definitions. Slices show a representative snapshot of each ROI and the corresponding z-coordinate in MNI space. ROIs were defined functionally and reflect activation that was greater for redirect than no-step trials across all the participants. (B) The full model used in the DCM analysis, with connections turned “on” displayed. The DCM model included connections between regions (solid arrows), inhibitory self-connections (dot tipped arrows), and driving input (grey arrows with dashed lines). Modulation was modeled on all between region connections and inhibitory self-connections. (SEF: Supplementary Eye Field (bilateral); FEF: Frontal Eye Fields (bilateral); IFC: Interior Frontal Cortex; Thal: Thalamus; CD: Caudate; SC: Superior Colliculus).

To examine group differences in the contrasts outlined above, we used an ROI analysis approach in three cortical regions (FEF, SEF, and IFC) and three subcortical regions (SC, caudate, and thalamus), involved in the reactive inhibition of saccades in humans and non-human primates (see Fig. 2**A**). Cortical ROIs were guided by anatomical knowledge and defined functionally based on activation in the combined sample for the redirect versus no-step contrast thresholded at uncorrected p-value (p < 5 × 10^−8^; a threshold at which the FEF and IFC formed two separate clusters in the right hemisphere). FEF activation was observed bilaterally and these two clusters were treated as a single ROI during signal extraction. A single cluster encompassed bilateral SEF. Although the maximally activated cluster here was in the SEF, the cluster did extend slightly into the anterior cingulate cortex. IFC activation was only observed in the right hemisphere, consistent with prior reports (Thakkar et al., 2014). These clusters were dilated by one voxel to accommodate regional heterogeneity. Subcortical ROIs were defined anatomically and were manually delineated on the averaged, normalized high-resolution T1 images from an independent group of 37 healthy controls, as reported in Thakkar et al. (2014). Because normalization procedures are very effective in subcortical regions, each of these structures was clearly visible. Because we did not have any hypotheses regarding hemispheric lateralization of cognitive control functions in these subcortical regions based on our previous work (Thakkar et al., 2014), the ROIs were combined across hemispheres (see Supplementary Material*s* for an analysis of activation in the hemispheres separately). Local percentage signal change was extracted from each of these six ROIs for redirect and no-step trials. For each ROI, repeated-measures ANOVAs were conducted to investigate the effects of condition (redirect versus no-step), group, and their interaction. Significant group-by-condition interactions were followed up with independent t-tests to investigate group differences in each condition as well as paired t-tests to investigate effects of condition in each group.

#### 2.6.5 Dynamic causal modeling

In order to assess effective connectivity between our ROIs we used the DCM framework (Friston et al., 2016, Zeidman et al., 2019a, Zeidman et al., 2019b; DCM for fMRI using SPM12). This framework allowed us to examine whether the instruction to exert executive control over a planned movement modulated causal connections within and between regions comprising a proposed oculomotor control network.

DCM allows the investigation of causal influence between brain regions by building generative models of predicted neural activity (Friston et al., 2003, Zeidman et al., 2019a) and optimizing across a parameter space characterizing effective connectivity. These parameters reflect the effective connections between and within regions as well as the modulation of those connections based on the conditions in the task. First, a forward generative model is created by inferring neural activity underlying a BOLD response given driving input from the task, influence from connected regions, self-connections, and modulation by experimental conditions. Then, based on the inferred neural activity, a predicted BOLD signal is generated and compared to the measured time series. This predicted BOLD signal is iteratively optimized by adjusting parameters in the generative model. This optimization (inversion) balances the fit (between the predicted response and the observed time series) and the complexity (in terms of the change between each prior parameter value and the posterior estimated parameter value) of the model. By optimizing across these parameters, the dynamic causal influences of neural networks can be identified.

Here, we use this computational theoretical framework to examine similarities and differences between SZP and HC in effective connectivity within our proposed oculomotor control network (including FEF, SEF, right IFC, caudate, thalamus, and SC). For each of the ROIs from the GLM analysis, we identified the peak t-statistic from the redirect > no-step contrast, and an 8 mm radius sphere, inclusively masked by the larger ROI used in the GLM analysis, was generated around this peak. For bilateral ROIs, the masked spheres in each hemisphere were combined into a single mask, and the time series reflecting the primary eigenvector adjusted for the effects of interest was extracted for each individual (see Supplementary Material*s* for an analysis of activation in redirect and no-step conditions within these individually-defined ROIs). In line with published recommendations (Zeidman et al., 2019a), we set the slice timing model in our DCM to half the TR. The network connectivity (see Fig. 2**B**) was based on anatomical connections between regions known to show saccade related physiology in the animal literature (e.g., FEF, SEF, SC), as well as regions known to be involved in reactive inhibition of planned movements (e.g., rIFC and caudate) where the thalamus serves as a central hub between these regions. Based on the connections identified in the introduction between these regions nineteen of the 30 possible between-region connections, were switched “on” (solid arrows in Fig. 2**B**); whereas other between-region connections, deemed to be biologically unlikely, were switched “off”. In order to properly model excitatory / inhibitory balance within each region, self-connections reflecting self-inhibition in each region were also switched “on”. A new GLM was created for the purpose of defining onsets to the DCM model with a regressor for task – all events in the experiment (no-step, compensated, noncompensated, and follow trials) – that were modeled as driving input to all locations (dashed arrows in Fig. 2**B**). A second regressor for redirect trials (both compensated and noncompensated trials) was defined to exert modulation related to reactive inhibition on all between-region and within-region connections. The input was mean centered with inputs scaled to account for a zero duration (see Zeidman et al., 2019a), which means that between-region and within-region connections (instantiated in an “A matrix”) should be interpreted as the average effective connectivity and modulation parameters (instantiated in a “B matrix”) add or subtract from that average. An exploratory analysis that examined modulation related to successful inhibition, with regressors for task and compensated trials to examine modulation of effective connectivity on compensated redirect trials, is presented in Supplementary Material*.*

In order to optimize our search space of models for each participant we inverted a full model for each participant and used a Parametric Empirical Bayes (PEB) analysis (optimized over the A and B matrices), followed by Bayesian Model Comparison (using the function spm_dcm_peb_bmc), to estimate average parameter values for each group (Friston et al., 2016, Zeidman et al., 2019a, Zeidman et al., 2019b). This approach provided parameter estimates averaged across possible models and weighted by model evidence. The approach identified two results for each group: the mean effective connectivity within the task (A matrix) and the modulation due to the instruction to redirect the planned saccade (B matrix). This allowed us to characterize how effective connectivity differentially contributes to executive control over saccade inhibition within each group.

Next, we used a second-level PEB to assess commonalities and differences between the group models. We constructed this second-level PEB twice – once as a PEB composed of the group PEBs and once as a single PEB with all participant’s inverted DCM models. The free energy of these two second-level models were compared (F_singlePEB_ = −39058; F_PEBofPEBs_ = −39113) and the single PEB with all participants was found to have higher free energy – therefore this modeling approach was used. Again, Bayesian Model Averaging was used to summarize parameter estimates. This yielded four results: overall mean effective connectivity (A matrix) across both groups, mean modulation (B matrix) across both groups, group differences in mean effective connectivity (differences in A matrices), and group differences in mean modulation due to the instruction to redirect (differences in B matrices).

Our interpretation of the results of the DCM modeling focuses on parameters with greater than 95% posterior probability (labeled as “credible” from here onwards). Parameters in the mean effective connectivity analysis with credible positive values (“excitatory” connections) show effective connectivity where increased activity in the source region leads to increased change in activation in the receiving region. Credible negative parameter values (“inhibitory” connections) show effective connectivity such that increased activity in the source region cause decreased changes in activation in the receiving region. Self-connections reflect within-region inhibition where the initial (default) parameter value starts at −0.5 and positive parameters reflect more inhibition whereas negative connections reflect less self-inhibition than this starting value. Modulation parameters reflect additive changes in effective connectivity on redirect trials relative to the mean effective connectivity throughout the modeled events.

Finally, we investigated putative relationships between oculomotor control network effective connectivity and both TSRT (in both groups) and occupational functioning measured with the SFS employment subscale (in SZP only). Parameters from individual optimized DCM models returned from the first level PEB analysis were extracted from all connections with credible group differences (both mean effective connectivity and modulation on redirect trials) in the second level PEB analysis. These parameters were put into backwards stepwise regressions in R (R development core team, 2013). Then, iteratively, non-significant parameters were removed until model fit was not improved by removing an additional factor. To predict TSRT, we followed the same stepwise elimination procedure; however, we maintained a persistent factor of group in each step of the parameter elimination process in order to model group differences in TSRT.

## 3 Results

### 3.1 Behavioral data

See Table 2 for summary of behavioral data.

**Table 2:** Search-step task performance.

	HC mean (s.d.)	SZP mean (s.d)
No-Step Reaction Time (ms)	322.51 (76.68)	312.34 (62.18)
Compensated Reaction Time (ms)	312.04 (58.44)	311.87 (65.65)
Noncompensated Reaction Time (ms)	290.58 (47.30)	270.66 (31.92)
TSRT (ms)	158.74 (19.30)	170.51 (27.32)

HC, healthy control subjects; SZP, persons with schizophrenia; TSRT: target step reaction time

#### 3.1.1 Probability of inhibition

The dynamic tracking procedure was successful at ensuring that participants failed to compensate for the target jump on approximately half of the redirect trials. The mean percentage of noncompensated trials was 47% and there was no group difference (t(43) = 0.34, p = 0.74).

#### 3.1.2 Speed of response execution

**Fig. 3:**
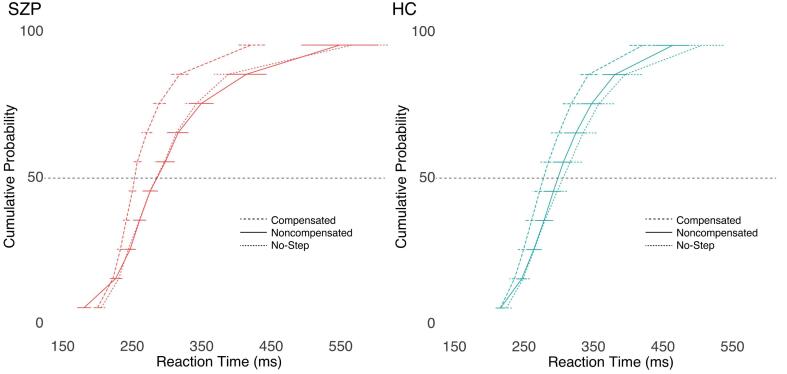
Vincentized reaction time distributions for no-step trials (fine dashed lines), compensated trials (relative to T2 onset; wide dashed lines), and noncompensated trials (solid lines). For each subject, RTs for each of the three trial types were binned into deciles. Decile means were averaged across subjects within SZP (left) and HC (right) to create the group-averaged reaction time distributions. Error bars indicate standard error of the mean. HC, healthy controls; SZP, persons with schizophrenia.

Cumulative RT distributions are presented in Fig. 3. There was a significant effect of trial type on RT (F(1.97, 84.58) = 17.16, p < 0.001, η^2^ = 0.07). Follow-up paired t-tests were conducted. Consistent with race model logic, non-compensated trials were significantly faster than both no-step (t(44) = 5.68, p < 0.001) and compensated (t(44) = 4.23, p < 0.001) trials. There was no difference between RTs on no-step and compensated trials (t(44) = 0.88, p = 0.385). Furthermore, there was no significant main effect of group (F(1,43) = 0.41, p = 0.527, η^2^ = 0.01), nor group-by-trial type interaction (F(1.97,84.58) = 1.06, p = 0.350, η^2^ < 0.01).

#### 3.1.3 TSRt

**Fig. 4:**
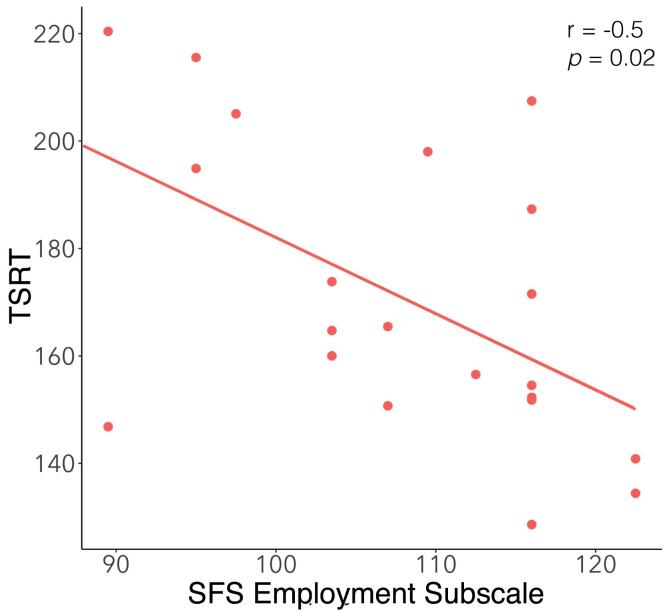
The relationship between the Social Functioning Scale employment subscale and TSRT (target step reaction time).

TSRT was significantly longer in SZP than in HC (t(43) = 1.68, p = 0.050). In addition, slower TSRT was related to lower SFS employment subscale scores (r_s_ = −0.50, p = 0.02), indicating that poorer occupational functioning was related to more inefficient inhibition (see Fig. 4).

### 3.2 fMRI data

#### 3.2.1 General linear modelling

**Fig. 5:**
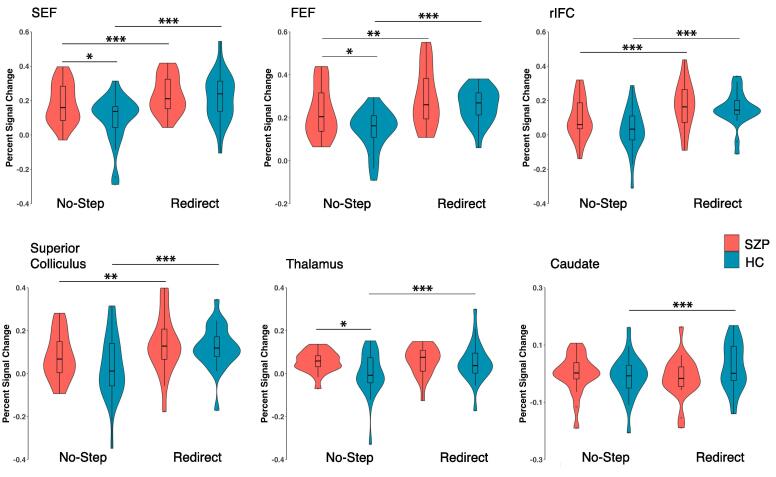
Violin plot of percent signal change for no-step and redirect trials in the six ROIs for healthy controls and persons with schizophrenia. Boxplots show the median and the four quartiles, where outliers beyond two interquartile intervals from the median are shown as individual dots. Significant differences visualized reflect paired t-tests for within group comparisons and independent t-tests for between group comparisons (p < 0.05*, p < 0.01**, p < 0.001***).

Results from ROI analyses are depicted in Fig. 5. There was a significant effect of condition in all cortical ROIs (all p’s < 0.001), which was expected as these regions were identified as those that showed a main effect of condition at the whole-brain level. Paired t-tests revealed that redirect trials elicited significantly greater activation than no-step trials within both groups in all three cortical regions: bilateral SEF (HC: t(23) = 6.46, p < 0.001; SZP: t(20) = 4.29, p < 0.001), bilateral FEF (HC: t(23) = 8.72, p < 0.001; SZP: t(20) = 3.79, p = 0.001), and right IFC (HC: t(23) = 7.70, p < 0.001; SZP: t(20) = 5.72, p < 0.001). There were no significant main effects of group in any cortical regions (bilateral FEF: F(1,43) = 3.75, p = 0.059, η^2^ = 0.07; bilateral SEF: F(1,43) = 2.13, p = 0.152 η^2^ = 0.04; right IFC (F(1,43) = 1.46, p = 0.234 ,η^2^ = 0.03). However, all three cortical regions showed significant group-by-condition interactions: bilateral FEF (F(1,43) = 5.03, p = 0.030, η^2^ = 0.01), bilateral SEF (F(1,43) = 9.30, p = 0.004, η^2^ = 0.02), and right IFC (F(1,43) = 4.68, p = 0.036, η^2^ = 0.01). These interaction effects were largely driven by increased activation in the no-step condition in SZP relative to HC and no group difference in activation in the redirect trials, resulting in reduced differential activation between redirect and no-step trials in SZP. SZP had greater activation than HC for no-step trials in the SEF (t(43) = 2.27, p = 0.028) and FEF (t(43) = 2.62, p = 0.012), but not for redirect trials in the SEF: (t(43) = 0.47, p = 0.638) or FEF: (t(43) = 1.07, p = 0.291). The IFC showed a similar pattern although differences between SZP and HC did not reach significance for either no-step (t(43) = 1.74, p = 0.088) or redirect (t(43) = 0.52, p = 0.602) trials.

In our subcortical ROIs, we saw no significant main effects of group (all p’s greater than 0.171). We found a significant main effect of condition, with greater activation on redirect than no-step trials, in the SC (F(1,43) = 31.52, p < 0.001, η^2^ = 0.08) and the thalamus (F(1,43) = 11.22, p = 0.002, η^2^ = 0.03), but not the caudate (F(1,43) = 1.53, p = 0.223, η^2^ = 0.01). These main effects were qualified by group-by-condition interaction effects in the caudate (F(1,43) = 7.91, p = 0.007, η^2^ = 0.03) and the thalamus (F(1,43) = 7.65, p = 0.008, η^2^ = 0.02), but not in the SC (F(1,43) = 1.95, p = 0.170, η^2^ = 0.01). Paired t-tests revealed greater activation on redirect than no-step trials in the caudate and thalamus that were significant in HC (thalamus: t(23) = 4.46, p < 0.001; caudate: t(23) = 2.85, p = 0.009), but not SZP (thalamus: t(20) = −0.40, p = 0.693; caudate: t(20) = −1.13, p = 0.270). The interaction in the thalamus followed a similar pattern to the cortical ROIs with greater percent signal change in the no-step condition for SZP than HC (t(43) = 2.25, p = 0.030) and no group differences in the redirect condition (t(43) = −0.37, p = 0.713). The caudate showed no group differences for either redirect (t(43) = −1.66, p = 0.104) or no-step trials (t(43) = 0.42, p = 0.680).

Results from exploratory whole-brain analyses are described in Supplementary Material.

#### 3.2.2 Dynamic causal modeling

**Fig. 6:**
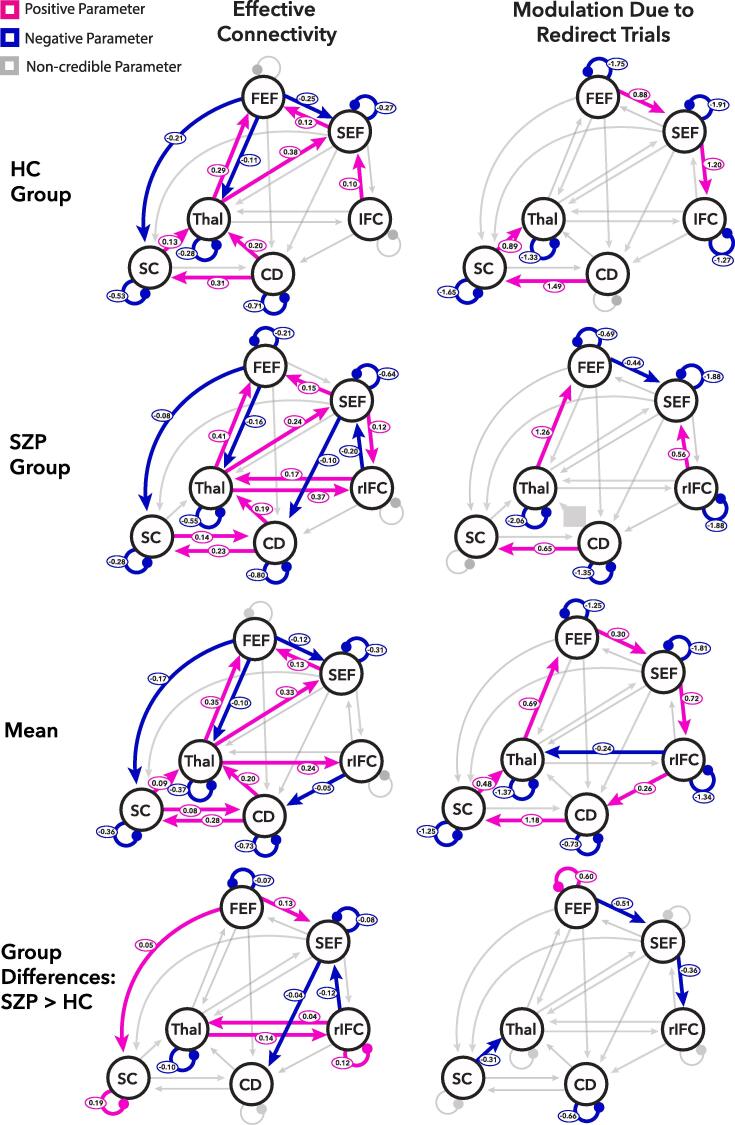
Dynamic causal modelling (DCM) results. Results from Bayesian model comparison of parametric empirical Bayesian analyses for healthy controls (HC) and persons with schizophrenia (SZP) are depicted on the top two rows. The results of the Bayesian model comparison of the second-level parametric empirical Bayesian analysis identifying group commonalities and differences are presented on the bottom two rows. Effective Connectivity (left column) refers to mean effects during all task conditions (A matrix) whereas Modulation Due to Redirect Trials (right column) refers to parameters additively modulating the mean activity by the requirement to inhibit and redirect a planned saccade to a new location (B Matrix). The Mean row reflects the group average effective connectivity and average modulation across both groups. The Group Differences row reflects connections with credible differences between groups. Parameters with posterior probability of being different from zero of greater than 95% are labeled with the corresponding parameter value and are color-coded based on the direction of effect.

Fig. 6 shows the mean effective connectivity between regions (left column) and modulation of that effective connectivity given the instruction to redirect (right column) for HC and SZP separately (top two rows), the mean across groups (third row), and credible between group differences (bottom row). All parameter estimates and their associated posterior probabilities are presented in *Supplementary Tables S1, S2, and S3***.** Description of the results below focuses on the Bayesian model comparison of the second-level PEB analysis (bottom two rows) representing group commonalities and group differences.

Collapsed across all groups, the shared mean effective connectivity (third row, first column of Fig. 6) largely recapitulates the network of interconnections known to be involved in saccade motor planning and execution. We see inhibitory effects from the FEF to the superior colliculus, thalamus, and SEF as well as from the IFC to the caudate. We see excitatory connectivity from the thalamus to FEF, SEF, and IFC, from the SEF to FEF, from the caudate to thalamus and to the superior colliculus, and from the superior colliculus to thalamus and caudate. Less self-inhibition than the initial default value was seen in all regions except FEF and IFC. Across groups, the instruction to redirect (third row, second column of Fig. 6) modulated self-connections within all regions, whereby it reduced the degree of self-inhibition. The common modulation associated with the instruction to redirect increased the inhibitory influence from the IFC to the thalamus. The need to inhibit the saccade also increased excitatory connections from the FEF to the SEF, from the SEF to the IFC, from the IFC to the caudate, from the caudate to the SC, from the superior colliculus to the thalamus, and from the thalamus to the FEF.

The group-difference results from the second-level PEB (fourth row, Fig. 6) shows that overall, in the task, SZP showed less self-inhibition in the FEF, SEF, and thalamus, compared to HC. The superior colliculus and IFC showed more self-inhibition among SZP. Further, the FEF showed inhibitory effects on the SEF and superior colliculus in HC, but these connections were less inhibitory among SZP, resulting in higher mean connectivity in SZP. SZP showed more bidirectional excitatory effective connectivity between the thalamus and the IFC than HC, where these connections did not differ from zero. The IFC also showed excitatory connections to SEF in HC but inhibited the SEF in SZP, resulting in lower mean connectivity from IFC to SEF in SZP. The connection between SEF and caudate did not differ from zero in HC, but showed inhibitory effective connectivity in SZP, resulting in group differences. Next, SZP and HC showed differences in the degree to which the instruction to redirect a saccade modulated effective connectivity (fourth row, second column, Fig. 6**)**. SZP further inhibited the FEF to SEF connections given the instruction to redirect, but these modulations were excitatory in HC. In addition, HC showed excitatory modulation of the SEF to IFC connection, but SZP did not; thus, SZP had significantly smaller modulation of the SEF to IFC connection by the instruction to redirect. Moreover, HC showed excitatory modulation of the connection between the superior colliculus and thalamus on redirect trials whereas SZP did not, which was a significant group difference. Finally, there were also group differences in the way the instruction to redirect modulated self-inhibition. SZP showed more modulation related self-inhibition in the FEF than HC and less modulation related self-inhibition in the caudate than HC on redirect trials.

Our final aim here was to identify relationships between the strength of effective connectivity parameters within this oculomotor control network, TSRT (in both groups), and occupational functioning (among SZP only). For each individual, we took the mean effective connectivity and modulation parameters from their optimized model returned by the first-level PEB from each connection identified as credibly different between groups in the second-level PEB. These parameter estimates were used to predict scores on the employment subscale of the SFS in SZP using stepwise backwards elimination. Models predicting TSRT did not achieve a significant fit during the backwards elimination procedure. The set of parameters that differed between groups were also used to predict scores on the SFS employment scale among SZP. The combination of six mean effective connectivity and two modulation parameters significantly predicted employment (F(2,42) = 5.23, p = 0.012, adjusted R^2^ = 0.67; see Table 3). More positive mean between-region connectivity from SEF to FEF and from FEF to SC were associated with higher SFS employment scores, as was more self-inhibition in the FEF and more modulation of self-inhibition in the caudate. More positive reciprocal connections between thalamus and IFC were associated with lower employment scores, as were thalamic self-inhibition and modulation of FEF self-inhibition on redirect trials.

**Table 3:** Final regression model from the backwards elimination procedure predicting employment among SZP using DCM parameters showing credible group differences.

Fixed Effects	Estimate	Std. Error	T-Stat	P-value
Intercept	−322.49	222.65	−1.45	0.181
FEF self-inhibition	1344.48	380.64	3.53	0.006
SEF to FEF	112.45	29.12	3.86	0.004
FEF to SC	223.31	51.69	4.32	0.002
Thal self-inhibition	−2924.85	744.25	−3.93	0.003
Thal to IFC	−144.55	28.38	−5.09	<0.001
IFC to Thal	−974.00	170.43	−5.72	<0.001
Modulation of FEF self-inhibition	−184.87	40.99	−4.51	0.001
Modulation of CD self-inhibition	470.03	85.98	5.47	<0.001

Note: Thal refers to thalamus, CD refers to caudate, SC refers to superior colliculus, FEF refers to frontal eye fields, SEF refers to supplementary eye fields, IFC refers to inferior frontal cortex.

## 4 Discussion

The ability to inhibit planned actions given changing environmental contexts is a critical cognitive function—one that has been found to be impaired in individuals with schizophrenia. Here we explored the neural mechanisms of these impairments. Specifically, we used an oculomotor search-step task to identify group differences in efficiency of response inhibition, functional activity, and effective connectivity within a network of brain regions associated with saccade planning, execution, and inhibition. Several main findings emerged. Behaviorally, we found longer TSRT in individuals with schizophrenia, which related to poorer occupational functioning, attesting to the clinical relevance of these findings. Patterns of brain activation in several cortical and subcortical oculomotor control regions revealed that, compared to controls, individuals with schizophrenia had reduced differential activity in several oculomotor control regions between trials that required saccades to be inhibited and redirected versus those trials in which the subject executed a visually guided saccade. Finally, we found widespread group differences in effective connectivity within this oculomotor control network across the task and more limited group differences in how effective connectivity was modulated by the need to inhibit and redirect gaze. These results, which are discussed in turn, provide new insights into the neural mechanisms of inefficient inhibitory control in individuals with schizophrenia.

Current findings of longer TSRT replicate previous reports in individuals with schizophrenia (Hoptman et al., 2018, Huddy et al., 2009, Hutton et al., 2004, Nolan et al., 2011, Shin et al., 2013, Thakkar et al., 2011, Thakkar et al., 2015b, Tsujii et al., 2018, Van Voorhis et al., 2019). Interestingly, the relationship between SSRT/TSRT and employment in individuals with schizophrenia has now been replicated in three largely independent samples (Thakkar et al., 2011, Thakkar et al., 2015b), thus providing compelling evidence that either slower response inhibition has downstream behavioral implications that are relevant for successful employment, or, relatedly, that the putative alterations in brain dynamics that lead to slower response inhibition have broader cognitive consequences that bear on one’s pursuit or attainment of gainful employment. Bayesian cognitive modeling has suggested that longer SSRT/TSRT in schizophrenia is due largely to a failure to initiate the STOP process and partly to a slower initiation of the STOP process, rather than to an impairment in the inhibitory process as such (Hughes et al., 2012, Matzke et al., 2017). Thus, longer SSRT/TSRT may be best interpreted as reflecting attentional deficits related to the processing of behavioral cues. We would argue that this interpretation is in line with our fMRI data.

Longer TSRT in individuals with schizophrenia was accompanied by altered patterns of brain activation and connectivity. We identified reduced neural responses in individuals with schizophrenia relative to healthy controls for the redirect versus no-step contrast in the FEF, SEF, IFC, SC, and thalamus—key nodes in a network involved in oculomotor control. These results align with fMRI findings from antisaccade (Baran et al., 2016, Camchong et al., 2008, Dyckman et al., 2011, McDowell et al., 2002, Raemaekers et al., 2002, Tu et al., 2006), go/no-go (Fryer et al., 2019, Rubia et al., 2001), and stop-signal studies (Hughes et al., 2012, Zandbelt et al., 2011), which report a smaller difference in activation between movement execution and inhibition conditions in individuals with schizophrenia relative to healthy controls. In the current work, the reduced differential activation between redirect and no-step trials in individuals with schizophrenia was driven by individuals with schizophrenia having greater activation than healthy controls on no-step trials (as compared to active fixation) but comparable activity on redirect trials. That is, group activation differences were evident during going, but not stopping. Similar results where individuals with schizophrenia showed more activity than healthy controls in saccade conditions relative to inhibition conditions were observed by Fukumoto-Motoshita et al. (2009) and colleagues using the anti-saccade task and by Ford et al. (2004) in a go/no-go task. Nevertheless, the pattern of differential activation we observed was unexpected given the bulk of prior work (Baran et al., 2016, Camchong et al., 2008, Dyckman et al., 2011, Fryer et al., 2019, McDowell et al., 2002, Raemaekers et al., 2002, Tu et al., 2006) and not necessarily consistent with an abnormality in inhibitory processes, per se.

Interpreting the significance of greater activation within our ROIs on no-step trials in individuals with schizophrenia is complicated by the heterogeneity of neuronal populations and function of the oculomotor control network nodes as well as the various cognitive processes that may be operating. More specifically, greater activation may be related to group differences in target selection, movement preparation/execution (i.e., subthreshold movement activity related to competing saccade targets), or to inhibitory processes that may be inappropriately engaged on these trials. Arguing against the explanation that greater activation on no-step trials in individuals with schizophrenia is related to target selection or movement preparation are previous reports of *less* activation in individuals with schizophrenia when making visually-guided saccades outside of a more complex cognitive task (e.g., Keedy et al., 2006) and *less* activation related to detection of a salient visual target (Silverstein et al., 2009). Furthermore, if group differences in target selection and movement preparation explained greater activation in individuals with schizophrenia on no-step trials, we would expect to only find activation differences on no-step trials in regions that we know from monkey neurophysiology studies modulate significantly with visual input and movement execution and/or in which controls show significant activation on no-step trials compared to fixation. This was not the case: compared to healthy controls, individuals with schizophrenia showed group-by-condition interactions that were largely driven by group differences on no-step trials even in regions that are not traditionally associated with bottom-up target selection or saccade execution (e.g., IFC) and in regions in which controls did not show significant modulation of activation on no-step trials (e.g., caudate, thalamus). Instead, we considered a broader explanation that individuals with schizophrenia may show abnormalities in how the likelihood of the need to exert cognitive control over a planned action is computed and the way in which behavior is adjusted accordingly (as in: Harle et al., 2014, Harlé et al., 2015). These differences may result in longer TSRT (due to an abnormality in the way in which the inhibitory process is triggered) and a pattern of brain activation that is less specific to current task demands (i.e., in which compensated and no-step trials look more similar in individuals with schizophrenia than healthy controls).

This notion that patterns of brain activity and longer TSRT reflect a fundamental difference in the way that individuals with schizophrenia are engaging in the task, rather than being related to a specific inhibitory impairment, are bolstered by our effective connectivity analyses. We interpret the effective connectivity results by first considering how the nodes in our specified network interact to support the control of saccades. In neurophysiology studies of non-human primates performing the stop-signal task, movement neurons in the FEF and SC attenuate their firing upon presentation of stop-signal (Brown et al., 2008, Paré and Hanes, 2003). This modulation occurs before SSRT (Hanes et al., 1998, Paré and Hanes, 2003) and is thus argued to play a direct role in the control of eye movements. How exactly these movement neurons come to be modulated remains an open question. One possibility is via inhibitory interactions between movement and fixation neurons within the FEF and SC; indeed, fixation neurons increase their firing rate upon stop-signal presentation (Hanes et al., 1998, Paré and Hanes, 2003). The relative activation between these competing populations provides proximal mechanisms to inhibit saccades (Goffart et al., 2012, Izawa and Suzuki, 2020, Izawa et al., 2005). Alternatively, modulation of movement neurons may be spurred by signals from outside of the FEF and SC—for example, via signals from the basal ganglia (Hikosaka et al., 2000). The basal ganglia plays a major role in inhibition and is classically understood to inhibit movements through the so-called indirect pathway (reviewed in Hikosaka et al., 2000; although see Simonyan, 2019) that projects from the striatum—the primary input area of the basal ganglia – to the substantia nigra pars reticulata (SNpr), which increases its inhibition on SC directly, and influences cortical movement areas (e.g., FEF and SEF) via the thalamus. On the basis of human neuroimaging and neurostimulation work (Cai et al., 2012, Zandbelt et al., 2013), inputs to the striatum (Zandbelt & Vink, 2010) or subthalamic nucleus (Chen et al., 2020) of the basal ganglia from both the right IFC and supplementary motor complex have been argued to be central to the reactive inhibition of movement (Aron et al., 2016). Electrocorticographical recordings (Swann et al., 2012) and DCM analyses of fMRI data (Zhang & Iwaki, 2019) further suggest that the interactions between the supplementary motor complex and IFC may be instrumental in regulating inhibition by way of the basal ganglia. However, the IFC has also been implicated in more domain general roles such as attention or responses to infrequent events (Hampshire and Sharp, 2015, Wessel and Aron, 2017), and primate neurophysiology suggests that SEF activity is not modulated quickly enough to be directly involved in the reactive control of that movement (Huerta & Kaas, 1990). Instead, the SEF may proactively regulate the excitability of the saccade system (Stuphorn et al., 2010) based on recent performance, conflict between mutually incompatible responses, and the cost (or riskiness) of errors (Chen and Stuphorn, 2018, So and Stuphorn, 2010, So and Stuphorn, 2012, So and Stuphorn, 2016) via direct connections to the FEF (Huerta and Kaas, 1990, Schall et al., 1993) and SC (Huerta & Kaas, 1990) or via the basal ganglia (Nambu, 2004, Parthasarathy et al., 1992). In sum, there are several routes by which saccade-related activity in regions effecting the actions (SC and FEF) can be cancelled by activity in regions associated with inhibitory control (SEF, IFC, caudate) in the context of this task.

Our DCM analyses yielded group differences in both the mean effective connectivity and the degree to which effective connectivity was modulated by the instruction to redirect a saccade. We observed five credible group differences in modulation on redirect trials. First, we observed reduced self-inhibition in the caudate among individuals with schizophrenia on redirect trials. Healthy controls did not show modulation of caudate self-inhibition on redirect trials whereas individuals with schizophrenia showed reduced self-inhibition, which was related to poorer employment status. Neurophysiology studies of inhibitory control point to differential use of basal ganglia pathways as a possible mechanism through which behavioral differences in inhibitory control tasks might arise (Hikosaka et al., 2000, Nambu, 2004, Parthasarathy et al., 1992) and recent work suggests that action cancelation may rely on inhibition of striatal action plans (Mallet et al., 2016). Reduced self-inhibition within the caudate may therefore reflect reduced inhibition related to action cancelation signals that could arise from engagement of different basal-ganglia pathways across groups. Second, we observed that the instruction to redirect decreased self-inhibition in the FEF in both groups, but to a significantly lesser extent in SZP, which was also related to employment status. As noted above, inhibition of movement activity in the FEF may arise via interactions between movement and fixation neurons, which may be altered in SZP, thereby contributing to less effective or efficient movement inhibition. Findings that altered modulation of self-inhibition of both the caudate and FEF by the instruction to redirect a planned movement were related to employment status attest to the potential real-world functional implications of these altered network dynamics. Third, we observed relatively less excitation from superior colliculus to the thalamus on redirect trials in SZP as compared to HC, perhaps reflecting reduced cortical influence of bottom-up visual and movement plan information among patients, a signal that may facilitate updating action plans or registering changing target locations. Fourth, we observed less excitatory modulation among patients in the connection between SEF and IFC, a connection that may be important for conveying information regarding the likelihood that the targets will change location in order to monitor for relevant signals (Swann et al., 2012). Finally, we observed that individuals with schizophrenia show alterations in the degree to which the need to redirect modulates the connection from FEF to SEF. Modulation on redirect trials in the two groups showed opposite directions of effect where healthy controls showed more excitation but individuals with schizophrenia showed inhibition. We may consider one role of the projections from FEF to SEF as conveying the degree of conflict between mutually incompatible responses (i.e., between activity of movement and fixation neurons in the FEF), which the SEF can use to proactively bias the excitability of the saccade system (Stuphorn et al., 2010). Increased inhibitory modulation of the FEF-SEF and SEF-IFC connections may thus reflect altered engagement of proactive control processes on a trial-to-trial basis in individuals with schizophrenia. Indeed, individuals with schizophrenia have been found to make more exaggerated adjustments of reaction times on the basis of the previous trial: they slow down more than controls following a trial in which they have to exert control over action (Barton et al., 2005, Barton et al., 2006, Thakkar et al., 2011, Thakkar et al., 2015b).

In addition to the modulatory effective connectivity differences between groups on redirect trials, we also observed widespread group differences in average effective connectivity across the task. Four notable differences were found. First, within-region inhibition was altered in individuals with schizophrenia in the FEF, SEF, IFC, thalamus, and superior colliculus. These alterations may be consistent with evidence for altered inhibitory interneuron function and resulting excitatory / inhibitory imbalance, a proposed mechanism underlying schizophrenia (Anticevic and Lisman, 2017, Krystal et al., 2003) that could impair the fidelity of recurrent activity necessary to maintain goal and action representations in cortex (Fan and Hu, 2018, Murray et al., 2014, Murray and Wang, 2018). We also observed differences between groups in between-region connections. Individuals with schizophrenia showed more inhibitory effective connectivity between IFC, SEF, and caudate – a key network for engaging and effecting inhibitory control (Aron et al., 2016, Swann et al., 2012, Zhang and Iwaki, 2019). Additionally, we found thalamocortical differences in the form of increased excitatory connectivity between the thalamus and IFC among patients. These differences may reflect aberrant feedback to the frontal cortex – a finding in line with prior DCM findings in individuals at risk for or with schizophrenia (Diwadkar et al., 2014, Limongi et al., 2020). Finally, we also found that individuals with schizophrenia had reduced mean inhibitory influence from the FEF to the SEF and to the superior colliculus. This reduced inhibitory influence from the FEF may reflect less propagated information about activity in visual, movement, and fixation neurons in FEF, information that the SEF uses to assess trial-by-trial proactive control demands (Stuphorn, 2015) and that contributes to movement plans in the superior colliculus (Matsumoto et al., 2018). Broadly, the finding that effective connectivity across the task showed widespread alterations in individuals with schizophrenia aligns with our ROI analyses that revealed greater activity on no-step trials in individuals with schizophrenia compared to controls, resulting in reduced differential activity between redirect and no-step trials, and suggesting a broad alteration in task-related activity. Furthermore, the observed relationships between employment status and mean effective connectivity parameters again hints at the real-world implications of disruptions in this oculomotor control network.

There are a number of limitations to the current study that suggest the results should be seen as exploratory. First, the current sample of individuals with schizophrenia is relatively small, asymptomatic, and high-functioning. Thus, correlational analyses should be considered exploratory, and future studies should aim to replicate these results in a larger and more clinically heterogeneous sample. In addition, medication may have confounded group comparisons. Arguing against that, however, is our finding that normalized antipsychotic dose did not correlate with TSRT, activation differences between redirect and no-step conditions, or any DCM parameters showing credible group differences (see Supplementary Material). A further limitation is that we did not include some brain regions that are also likely involved directly or indirectly in oculomotor control, for example, additional subregions of the basal ganglia, the superior parietal lobe, and dorsolateral prefrontal cortex. A more complete network was not selected in an effort to reduce our model space (in the cases of the superior parietal lobe and dorsolateral prefrontal cortex) and due to the absence of a reliable signal in smaller, deep brain structures (in the case of subregions of the basal ganglia). A final limitation is that we only considered direct connections between nodes, rather than including indirect connections – where activity in one node influences the connection between two other nodes. This may be considered in future work using non-linear DCM (Stephan et al., 2008).

Despite these limitations, the current work provides new insights into the mechanisms of reactive control abnormalities in individuals with schizophrenia. Consistent with prior reports, individuals with schizophrenia show abnormalities in the reactive control of an action that is related to an important functional outcome: employment status. Brain activation patterns indicate abnormal activation of key regions in an oculomotor control network in schizophrenia patients. The effective connectivity analysis demonstrates how this network is functioning in schizophrenia and suggests that the need to rapidly inhibit and change a planned action may engage *different* pathways in individuals with schizophrenia. Inhibitory control for individuals with schizophrenia is clinically relevant given links to poor psychosocial outcomes (Barch and Sheffield, 2017, Bilder et al., 2000, Green et al., 2000, Nuechterlein et al., 2004, Shin et al., 2013). The current results fit into this larger literature, elaborating upon the neural mechanisms within the oculomotor control network that differ between groups, and that show relationships to real-world metrics. These findings lend themselves to a more precise understanding of altered dynamics within this network in individuals with schizophrenia engaging in inhibitory control and may, down the line, play a role in identifying neurostimulation targets that might ameliorate cognitive control.

## CRediT authorship contribution statement

**Matthew Lehet:** Conceptualization, Methodology, Software, Formal analysis, Writing - original draft, Visualization. **Ivy F. Tso:** Methodology. **Sebastiaan F.W. Neggers:** Conceptualization, Software, Resources, Funding acquisition, Supervision. **Ilse A. Thompson:** Investigation. **Beier Yao:** Methodology. **René S. Kahn:** Conceptualization, Resources, Funding acquisition, Supervision. **Katharine N. Thakkar:** Conceputalization, Funding acquisition, Supervision, Project administration, Methodology, Software.
