# Supplementary figures and images for: Altered effective connectivity within an oculomotor control network in individuals with schizophrenia
Source: Neuroimage Clin. 2021 Jul 14;31:102764. doi: 10.1016/j.nicl.2021.102764 (PMC8313596; doi:10.1016/j.nicl.2021.102764)

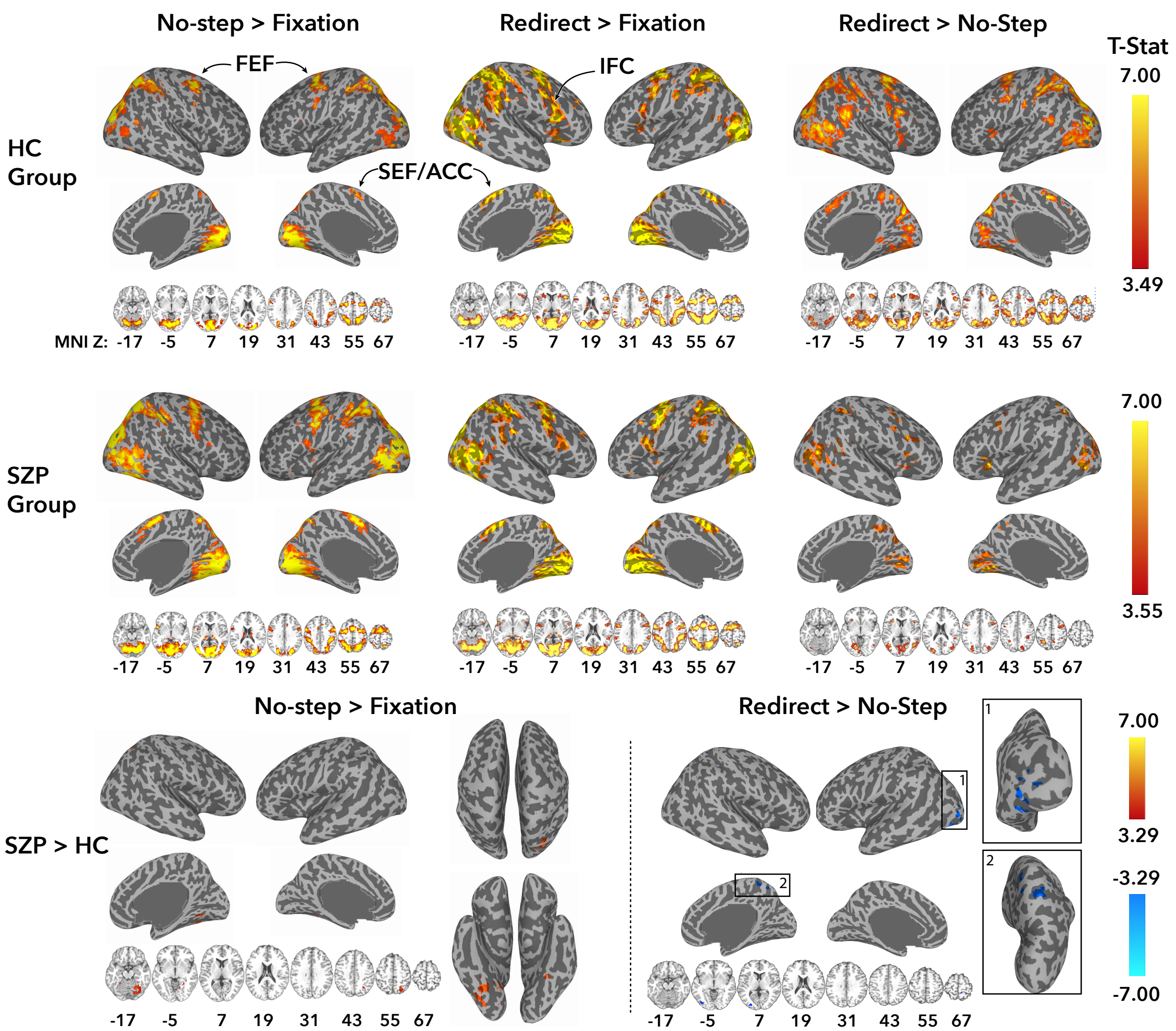

Supplement: Supplementary data 1 [file mmc1.pdf]
