# Supplementary material for: Altered effective connectivity within an oculomotor control network in individuals with schizophrenia
Source: Neuroimage Clin. 2021 Jul 14;31:102764. doi: 10.1016/j.nicl.2021.102764 (PMC8313596; doi:10.1016/j.nicl.2021.102764)
Supplement: Supplementary data 2 [file mmc2.pdf]

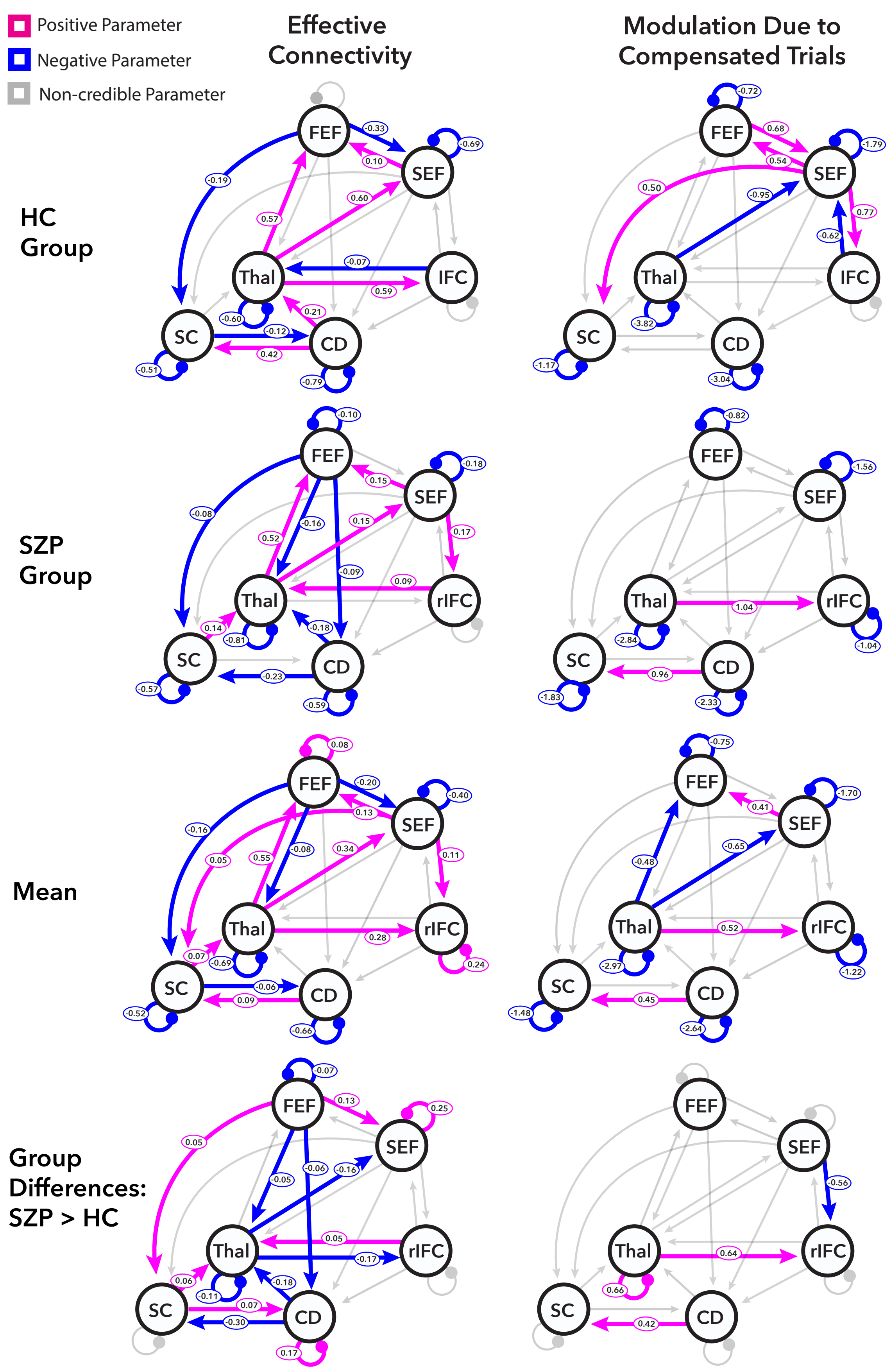

Positive Parameter
Negative Parameter
Non-credible Parameter
Effective Connectivity
Modulation Due to Compensated Trials
HC Group
SZP Group
Mean
Group Differences: SZP > HC
FEF
SEF
Thal
IFC
rIFC
SC
CD
