# Supplementary material for: Altered effective connectivity within an oculomotor control network in individuals with schizophrenia
Source: Neuroimage Clin. 2021 Jul 14;31:102764. doi: 10.1016/j.nicl.2021.102764 (PMC8313596; doi:10.1016/j.nicl.2021.102764)
Supplement: Supplementary data 3 [file mmc3.docx]

**Supplemental materials**

Altered effective connectivity within an oculomotor control network in individuals with schizophrenia

Lehet, M.^1^, Tso, I.F.^2^, Neggers, S.F.W.^3^, Thompson, I.A.^3^, Yao, B.^1^, Kahn, R.S.^3,4^, Thakkar, K.N.^1,3,5^

^1^Department of Psychology, Michigan State University, East Lansing, MI, USA

^2^ Department of Psychiatry, University of Michigan, Ann Arbor, MI, USA.

^3^ Department of Psychiatry, University Medical Center Utrecht, Utrecht, The Netherlands

^4^Department of Psychiatry, Icahn School of Medicine at Mount Sinai, New York, NY, USA

^5^Deparment of Psychiatry and Biobehavioral Medicine, Michigan State University, Grand Rapids, MI USA

Corresponding author: Katharine N. Thakkar, Ph.D., Department of Psychology, Michigan State University, 316 Physics Road, Room 110C, East Lansing, Michigan 48824. Phone: (517)884-8489. Email: [kthakkar@msu.edu](mailto:kthakkar@msu.edu)

**Activity on compensated versus noncompensated trials**

Methods

Within each ROI the activation associated with compensated and noncompensated trials was extracted and compared in order to assess the validity of collapsing across conditions in the main analysis.

Results

No regions showed significantly greater activation on compensated as compared to noncompensated trials using one tailed t-tests: SEF t(44) = -2.07, p = 0.978; FEF t(44) = -2.39, p = 0.989; rIFC t(44) = -1.53, p = 0.934; superior colliculus t(44) = -1.10, p = 0.862; thalamus t(44) = -1.04, p = 0.848; caudate t(44) = 0.69 p = 0.248. This suggests that trials where participants successfully inhibited a saccade did not recruit more inhibition-related activity than noncompensated trials. As we have argued previously, the difference between brain activity related to successfully inhibited saccades versus unsuccessfully noncompensated saccades might be more related to the timing than the magnitude of activation, which is not captured by the sluggish BOLD signal. We take this as support for our analytic approach of combining these trial types into a single condition.

**Exploratory Whole Brain Analysis**

Methods

We conducted an exploratory whole brain analysis, examining the contrasts described in the manuscript (redirect versus fixation, no-step versus fixation, and redirect versus no-step) using uncorrected voxel thresholds p < 0.001. Statistical maps were masked to include voxels within grey matter and significant clusters were defined based on p < 0.05 FWE-corrected for multiple cluster-level comparisons.

Results

Significant within-group activation clusters for the redirect versus no-step contrast are presented in **Table S1** and **Figure S1**. At a whole-brain level, HC showed activation in a large network of frontal, parietal, and occipital regions for redirect relative to no-step trials. Of particular interest were regions involved in saccade planning and execution such as bilateral FEF, bilateral SEF extending into right ACC, and right IFC for redirect versus no-step trials. In SZP, bilateral patches of mid occipital cortex and medial parietal cortex showed a significant difference in the redirect versus no-step contrast as well as less widespread FEF and right IFC activation relative to HCs. The direct comparison of groups revealed greater activation in HC than SZP in the redirect versus no-step contrast in left medial occipital cortex and right superior parietal cortex. There were no regions where SZP showed greater activation in the redirect versus no-step contrast than HC.

Examination of group differences in the redirect versus fixation and no-step versus fixation contrasts separately reveal that group differences in the redirect versus no-step contrast are driven by group activation differences on no-step trials (see **Figure S1**). SZP showed greater activation than HC for no-step trials in a number of regions including bilateral fusiform extending down into the cerebellum, right superior occipital cortex, and right superior parietal cortex. There were no areas where HC showed greater activation than SZP for no-step trials relative to fixation. No differences were found between groups in redirect versus fixation contrasts. Therefore, the group differences in the redirect versus no-step contrast are best explained by greater activation in the no-step condition for SZP.

**Hemispheric Analyses**

Methods

In order to ensure that combining subcortical and FEF ROIs across hemispheres did not obscure hemispheric specialization or differentiation, we assessed activation in the two hemispheres separately. Local percentage signal change was extracted from both right and left hemisphere for the thalamus, superior colliculus, caudate, and FEF for redirect and no-step trials. For each region, ANOVAs were conducted to investigate the within-subjects factors of condition (redirect versus no-step), and hemisphere, as well as the between subjects factor of group, including all two-way interactions and the three-way interaction. These analyses were performed on the ROIs that were used in the GLM analysis as well as on the individually-defined ROIs that were used in the DCM analysis.

Results

*Anatomical and functional ROIs defined at group level and used in GLM analysis*

Here, we only report the effects involving hemisphere. There was no significant main effect of hemisphere or interactions with hemisphere in the superior colliculus (all p-values > 0.071) or in the thalamus (all p-values > 0.206). In the caudate there was a significant main effect of hemisphere (F(1,43) = 9.54, p = 0.004, η^2^ = 0.01), with greater activation in the left hemisphere (left: mean=0.01, s.d.= 0.07; right: mean=-0.01, s.d.=0.08). However, there were no interactions involving hemisphere (all p-values>0.229). In the FEF, there was a marginal main effect of hemisphere (F(1,43) = 3.96, p = 0.053, η^2^ = 0.02), with greater activation in the left hemisphere, but there were no interactions involving hemisphere (all p-values>0.076).

*ROIs defined at the individual level and used in DCM analysis*

The superior colliculus, the thalamus, and the caudate all showed no main effects of hemisphere or interactions with hemisphere (all p-values >0.089). In the FEF, although there was no main effect of hemisphere F(1,43)=1.56, p=0.219, η^2^ = 0.01 ), there was a significant hemisphere-by-condition interaction (F(1,43)=13.62, p=0.001, η^2^ = 0.00). This interaction was driven by significantly greater activation between hemispheres on no-step (t(44) = -2.07, p = 0.045; left: mean = 0.21; sd = 0.13; right: mean = 0.16; sd = 0.15), but not redirect trials (t(44) = -0.55, p = 0.588; left: mean = 0.33; sd = 0.16; right: mean = 0.32; sd =0.17). Importantly, in both hemispheres, activation in the FEF was greater on redirect than no-step trials (left: t(44)= 9.28, p<0.001); right: t(44)=10.68; p<0.001).

**Redirect and no-step trial activation in the individually-defined ROIs for the DCM**

Methods

In order to characterize the variance that could be explained by the instruction to redirect gaze in the ROIs that were used in the DCM analysis, local percentage signal change was extracted from each of the six ROIs for redirect and no-step trials. For each ROI, repeated-measures ANOVAs were conducted to investigate the effects of condition (redirect versus no-step), group, and their interaction. Significant group-by-condition interactions were followed up with independent t-tests to investigate group differences in each condition as well as paired t-tests to investigate effects of condition in each group.

Results

There was a significant effect of condition in all cortical ROIs (all p’s<0.001), which was expected as these ROIs were sub-regions of those that showed a main effect of condition at the whole-brain level, and were centered around peak voxels in the redirect versus no-step contrast per person. Paired t-tests revealed that redirect trials elicited significantly greater activation than no-step trials within both groups in all three cortical regions: bilateral SEF (HC: t(23)=8.08, p<0.001; SZP: t(20)= 7.09, p<0.001), bilateral FEF (HC: t(23)= 10.83, p<0.001; SZP: t(20)= 5.47, p<0.001), and right IFC (HC: t(23)= 10.61, p<0.001; SZP: t(20)= 7.70, p<0.001). No main effects of group were seen in FEF and SEF (all ps>0.236; however, an interaction between group and condition was seen in the SEF (F(1,43) = 8.72, p = 0.005, η^2^ = 0.01) but not in the FEF (F(1,43) = 3.11, p = 0.085, η^2^ = 0.01) or in the IFC (F(1,43) = 3.34, p = 0.075, η^2^ = 0.01). As in the main results, this interaction was driven by reduced differences between redirect and no-step trials for SZP relative to HC in the SEF. Neither trial type showed significant differences between groups in follow-up t-tests; however, due to increased no-step activation among SZP, there was more difference between groups on no-step trials (t(43)=1.38,p=0.176) than on redirect trials (t(43)=-0.15,p=0.879).

In our subcortical ROIs, we also found a significant effect of condition in all regions (all p’s<0.001), which was expected as these ROIs were centered around peak voxels in the redirect versus no-step contrast per person, within the anatomically defined regions. Paired t-tests revealed that redirect trials elicited significantly greater activation than no-step trials within both groups in all three subcortical regions: bilateral caudate (HC: t(23)=6.15, p<0.001; SZP: t(20)= 4.41, p<0.001), bilateral thalamus (HC: t(23)= 8.25, p<0.001; SZP: t(20)= 3.67, p=0.002), and bilateral superior colliculus (HC: t(23)= 6.26, p<0.001; SZP: t(20)= 3.88, p=0.001). We found no main effects of group (all ps>0.304); however, interactions between group and condition were seen in the thalamus (F(1,43) = 12.99, p = 0.001, η^2^ = 0.02) but not in the caudate (F(1,43) = 3.42, p = 0.071, η^2^ = 0.01) or the superior colliculus (F(1,43) = 2.28, p = 0.139, η^2^ = 0.01). Follow up t-tests suggested that – as in the main results – the interaction in the thalamus was driven by SZP showing reduced differences between redirect and no-step trials relative to HC. This reduced difference was due to increased activation on no-step trials for SZP: no-step trials showed more difference between groups (thalamus: t(43)=1.82, p=0.075) than did redirect trials (t(43)=-0.05, p=0.953). These results suggest that the selection process for individual ROIs used in the DCM was able to capture variance in all ROIs that can be explained in the DCM analysis.

**DCM examining the modulatory effect of compensated trials**

Methods

In order to examine modulation associated only with successful inhibition and redirection of a planned saccade, we ran a version of the DCM from the main text that examined modulation on compensated trials instead of all redirect trials. The same ROIs and connections were used in both analyses, but the onsets submitted to the DCM consisted of conditions for task (including all trials of all types) and modulation was assessed on compensated redirect trials. Separate PEBs were calculated for the two groups. For the comparison between groups, a PEB composed of the two group PEBs and a single PEB including inverted DCMs from all participants were compared and the single PEB approach had better free energy so that model is reported here.

Results

**Figure S2** shows the mean effective connectivity between regions (left column) and modulation of that effective connectivity on compensated trials (right column) for HC and SZP separately (top two rows), the mean across groups (third row), and credible between group differences (bottom row). All parameter estimates and their associated posterior probabilities are presented in Supplementary **Tables S5**, **S6**, and **S7.** Description of the results below focuses on the Bayesian model comparison of the second-level PEB analysis (bottom two rows) representing group commonalities and group differences.

Collapsed across all groups, the shared mean effective connectivity (third row, first column of **Figure S2**) largely recapitulates the network of interconnections shown in the analysis with redirect modulation. The DCM framework predicts changes in activity based on both the mean and modulation connectivity using a generalized linear model. Therefore, changes to the specified modulation have the potential to alter the mean effective connectivity calculated in this analysis relative to the analysis in the main paper. We see inhibitory effects from the FEF on SC, thalamus, and SEF, and from superior colliculus to the caudate. We see excitatory connectivity from the thalamus to FEF, SEF, and IFC; from the SEF to IFC, to FEF, and to the superior colliculus; from superior colliculus to the thalamus; and from the caudate to the superior colliculus. Less self-inhibition than the initial default value was seen in the thalamus, caudate, superior colliculus, and SEF. More self-inhibition than the default was seen in the FEF and IFC. Across groups, successfully redirecting the planned saccade (third row, second column of **Figure S2**) modulated self-connections within all regions, whereby it reduced the degree of self-inhibition. The common modulation associated with successfully redirecting the planned saccade increased the excitatory connections from the SEF to the FEF, from the caudate to the SC, and from the thalamus to the IFC. Inhibitory modulation from the thalamus to the FEF and SEF was also seen. Common between this analysis and the model in the main text are decreased inhibition across regions and excitation from the caudate to the superior colliculus.

The group-difference results from the second-level PEB (fourth row, **Figure S2**) shows that overall, in the task, SZP showed less self-inhibition in the FEF and thalamus, compared to HC. The caudate and SEF showed more self-inhibition among SZP; however, this effect was still below the default self-inhibition. The FEF showed inhibitory effects on the SEF and superior colliculus in HC, but these connections were less inhibitory among SZP, resulting in more mean connectivity in SZP. Further, SZP showed more excitatory effective connectivity from the superior colliculus to the thalamus, and to the caudate. The IFC also showed excitatory influence on the thalamus in SZP whereas it was inhibitory in HC. The connection between the caudate and thalamus and between the caudate and superior colliculus were both inhibitory in SZP but excitatory in HC, leading to group differences. Connections from the thalamus to the SEF and IFC were more excitatory in HC than in SZP, which led to group differences. Finally, in the right column, SZP and HC showed differences in the degree to which correctly redirecting a saccade modulated effective connectivity. SZP inhibited the connection from SEF to IFC whereas there was excitatory modulation from thalamus to IFC and from caudate to superior colliculus. SZP also showed more self-inhibition in the thalamus than HC.

**Investigation of Medication as a Confounding factor**

Methods

In order to eliminate medication as a possible confounding factor we examined relationships between task performance, neural activation, effective connectivity parameters, and chlorpromazine (CPZ) equivalent antipsychotic dose in persons with schizophrenia using Spearman’s correlation coefficients. separate correlations were run comparing each measure with CPZ equivalent dose.

Results

There were no significant correlations found between normalized antipsychotic dose and any factor including TSRT (r_s_=0.11, p=0.667), employment (r_s_=0.43, p=0.079), differences between redirect and no-step in any ROI (all p-values>0.648), nor with any parameters that showed credible group difference in the DCM analysis (all p-values>0.255).

**Supplementary Figure Legends**

**Figure S1.** Visualization of GLM results for healthy controls (top row), patients (middle row), and the contrast between them (bottom row) for comparisons of no-step greater than fixation (left column), redirect greater than fixation (middle column), and redirect greater than no-step (right column). No areas showed significant effects for the between group comparisons in the redirect greater than fixation condition. Minimum T-statistics were set to an uncorrected p = 0.001. Only clusters that had a FWE corrected p-value p< .05 using cluster-wise multiple corrections are visualized. For the group comparisons warm colors reflect the contrast of patients greater than healthy controls whereas cool colors reflect healthy controls greater than patients. Slices show the same effects visualized at the labeled MNI Z axis slice number. Slices are visualized on a skull stripped MNI colin27 template using MRIcron whereas surface images are displayed on an MNI surface in Afni’s SUMA visualization tool.

**Figure S2.** Dynamic causal modelling (DCM) results. Results from Bayesian model comparison of parametric empirical Bayesian analyses for healthy controls (HC) and persons with schizophrenia (SZP) are depicted on the top two rows. The results of the Bayesian model comparison of the parametric empirical Bayesian analysis identifying group commonalities and differences are presented on the bottom two rows. Effective Connectivity (left column) refers to mean effects during all task conditions (A matrix) whereas Modulation Due to Compensated Trials (right column) refers to parameters additively modulating the mean activity when successfully inhibiting and redirecting a planned saccade to a new location (B Matrix). The Mean row reflects the group average effective connectivity and average modulation across both groups. The Group Differences row reflects connections with credible differences between groups. Parameters with posterior probability of being different from zero of greater than 95% are labeled with the corresponding parameter value and are color-coded based on the direction of effect.

**Supplementary Tables**

| Table S1. GLM results | | | | | | | |
| --- | --- | --- | --- | --- | --- | --- | --- |
|  | Cluster Size | Cluster wise | Peak | Peak MNI Coordinates (mm) | | |  |
| Analysis | (Voxels) | P (FWE-corr) | T-stat | x | y | z | Region |
| Controls: No-Step > Fixation | | | | | | | |
|  | 4721 | 0 | 14.84 | -6 | -78 | -3 | Left Lingual |
|  |  |  | 11.56 | 12 | -93 | 6 | Right Calcarine |
|  |  |  | 10.85 | 24 | -69 | -6 | Right Fusiform |
|  |  |  | 10.48 | -21 | -63 | 57 | Left Superior Parietal |
|  |  |  | 10.17 | 21 | -75 | -12 | Right Fusiform |
|  |  |  | 10.03 | -15 | -78 | -9 | Left Lingual |
|  |  |  | 9.87 | -9 | -96 | 12 | Left Superior Occipital |
|  |  |  | 9.77 | 12 | -96 | 15 | Left Superior Occipital |
|  |  |  | 9.66 | 21 | -90 | 18 | Right Cuneus |
|  |  |  | 9.42 | -33 | -66 | -12 | Right Superior Occipital |
|  | 366 | 0 | 8.82 | -24 | 0 | 54 | Left Superior Frontal |
|  |  |  | 7.46 | -45 | -3 | 54 | Left Precentral |
|  |  |  | 6.26 | -60 | 6 | 30 | Left Precentral |
|  |  |  | 4.01 | -48 | 0 | 39 | Left Precentral |
|  | 324 | 0 | 8.76 | 24 | -3 | 48 | Superior Frontal Gyrus |
|  |  |  | 7.02 | 42 | -3 | 51 | Right Precentral |
|  |  |  | 5.87 | 57 | 9 | 39 | Right Precentral |
|  |  |  | 4.04 | 48 | 9 | 51 | Right Precentral |
|  | 149 | 0 | 6.23 | 6 | 3 | 60 | Left Supplementary motor area |
|  |  |  | 5.77 | -3 | -3 | 66 | Left Supplementary motor area |
|  |  |  | 4.39 | 12 | 3 | 69 | Right Supplementary motor area |
| Patients: No-Step > Fixation | | | | | | | |
|  | 8223 | 0 | 12.5 | -12 | -66 | -21 | Left Cerebellum 6 |
|  |  |  | 12.36 | -3 | -72 | -24 | Vermis 7 |
|  |  |  | 12.22 | -3 | -78 | 0 | Left Lingual |
|  |  |  | 12.2 | 3 | -72 | -3 | Right Lingual |
|  |  |  | 12.01 | -6 | -81 | -9 | Left Lingual |
|  |  |  | 11.84 | 27 | -84 | 15 | Right Mid Occipital |
|  |  |  | 11.68 | 12 | -96 | 9 | Right Cuneus |
|  |  |  | 11.36 | 21 | -66 | 60 | Right Superior Parietal |
|  |  |  | 11.19 | -12 | -78 | 51 | Left Superior Parietal |
|  |  |  | 11.17 | 21 | -78 | -9 | Right Lingual |
|  | 1976 | 0 | 11.58 | -24 | -6 | 54 | Left Superior Frontal |
|  |  |  | 9.18 | 3 | 6 | 54 | Right Supplementary motor area |
|  |  |  | 8.98 | 27 | -3 | 57 | Right Mid Frontal |
|  |  |  | 8.76 | -51 | 3 | 36 | Left Precentral |
|  |  |  | 8.18 | -3 | 0 | 60 | Left Supplementary motor area |
|  |  |  | 7.96 | 57 | 3 | 42 | Right Precentral |
|  |  |  | 7.95 | -48 | 12 | -6 | Left Insula |
|  |  |  | 7.94 | -39 | -3 | 60 | Left Precentral |
|  |  |  | 7.36 | -51 | 0 | 45 | Left Precentral |
|  |  |  | 7.13 | -30 | -3 | 66 | Left Superior Frontal |
| Controls: Redirect > Fixation | | | | | | | |
|  | 9694 | 0 | 17.83 | -3 | -75 | 0 | Left Lingual |
|  |  |  | 16.61 | 12 | -90 | 6 | Right Calcarine |
|  |  |  | 15.27 | -27 | -78 | 21 | Left Middle Occipital |
|  |  |  | 14.3 | 24 | -66 | 54 | Right Superior Parietal |
|  |  |  | 14.2 | -18 | -78 | -12 | Left Calcarine |
|  |  |  | 13.93 | 39 | -48 | 51 | Right Inferior Parietal |
|  |  |  | 13.8 | 27 | -75 | -12 | Right Fusiform |
|  |  |  | 13.7 | 12 | -75 | -6 | Right Lingual |
|  |  |  | 13.46 | 18 | -75 | -12 | Right Lingual |
|  |  |  | 13.44 | 27 | -72 | 33 | Right Middle Occipital |
|  | 2511 | 0 | 14.03 | -24 | 0 | 54 | Left Superior Frontal |
|  |  |  | 13.87 | 30 | 0 | 57 | Right Middle Frontal |
|  |  |  | 9.15 | 54 | 12 | 33 | Right Precentral |
|  |  |  | 9.11 | 6 | 12 | 57 | Right Supplemental Motor Area |
|  |  |  | 8.94 | -36 | -3 | 51 | Left Precentral |
|  |  |  | 8.79 | 15 | 9 | 63 | Right Supplemental Motor Area |
|  |  |  | 8.76 | 42 | -3 | 48 | Right Precentral |
|  |  |  | 8.57 | 6 | 3 | 66 | Right Supplemental Motor Area |
|  |  |  | 8.53 | -30 | 0 | 66 | Left Superior Frontal |
|  |  |  | 8.42 | -3 | 9 | 51 | Left Supplemental Motor Area |
|  | 53 | 0.023 | 8.23 | -3 | -30 | -6 | Left Superior Colliculus |
|  |  |  | 7.66 | 6 | -30 | -6 | Right Superior Colliculus |
|  |  |  | 5.5 | 6 | -36 | -18 | Right Cerebelum |
|  |  |  | 3.76 | -3 | -36 | -18 | Vermis |
|  | 93 | 0.001 | 7.12 | 18 | -27 | 12 | Right Thalamus |
|  |  |  | 7.09 | 21 | -30 | 0 | Right Thalamus |
|  |  |  | 6 | 12 | -21 | 15 | Right Thalamus |
|  |  |  | 4.35 | 33 | -33 | 3 | Right Hippocampus |
|  | 108 | 0.001 | 6.64 | -21 | -33 | 9 | Left Hippocampus |
|  |  |  | 4.85 | -21 | -33 | -3 | Left Hippocampus |
|  |  |  | 4.79 | -15 | -9 | 18 | Left Thalamus |
|  | 79 | 0.004 | 6.44 | 42 | 42 | 33 | Right Middle Frontal |
|  |  |  | 5.45 | 39 | 36 | 24 | Right Middle Frontal |
| Patients: Redirect > Fixation | | | | | | | |
|  | 2443 | 0 | 7.15 | -24 | -3 | 57 | Left Superior Frontal |
|  |  |  | 6.11 | 3 | 9 | 54 | Right Supplemental Motor Area |
|  |  |  | 5.77 | -51 | 6 | 36 | Left Precentral |
|  |  |  | 5.69 | -51 | 0 | 45 | Left Precentral |
|  |  |  | 5.64 | 12 | 3 | 60 | Right Supplemental Motor Area |
|  |  |  | 5.54 | 33 | 0 | 48 | Right Precentral |
|  |  |  | 5.46 | -9 | -3 | 63 | Left Supplemental Motor Area |
|  |  |  | 5.42 | 30 | -3 | 57 | Right Middle Frontal |
|  |  |  | 5.36 | 45 | 9 | 39 | Right Precentral |
|  |  |  | 5.23 | 57 | 3 | 42 | Right Precentral |
|  | 9145 | 0 | 7.12 | -6 | -69 | 0 | Left Lingual |
|  |  |  | 6.8 | -3 | -78 | 0 | Left Lingual |
|  |  |  | 6.57 | 9 | -69 | 3 | Right Lingual |
|  |  |  | 6.53 | -6 | -81 | -9 | Left Lingual |
|  |  |  | 6.47 | -18 | -75 | -3 | Left Lingual |
|  |  |  | 6.43 | -3 | -72 | -21 | Vermis |
|  |  |  | 6.42 | 27 | -84 | 15 | Right Middle Occipital |
|  |  |  | 6.38 | 30 | -60 | -24 | Right Cerebelum |
|  |  |  | 6.36 | 27 | -75 | -15 | Right Fusiform |
|  |  |  | 6.32 | -24 | -69 | -21 | Left Cerebelum |
|  | 122 | 0 | 4.96 | -12 | -24 | 9 | Left Thalamus |
|  |  |  | 4.92 | -6 | -30 | -6 | Left Superior Colliculus |
|  |  |  | 4.63 | -18 | -30 | 0 | Left Thalamus |
|  |  |  | 4.3 | -27 | -27 | -3 | Left Hippocampus |
|  | 53 | 0.016 | 5.68 | 39 | 42 | 33 | Right Middle Frontal |
| Patients: Redirect > No-Step | | | | | | | |
|  | 69 | 0.002 | 7.87 | -30 | 21 | 3 | Left Insula |
|  |  |  | 5.34 | -36 | 18 | 9 | Left Insula |
|  | 634 | 0 | 7.39 | -15 | -78 | 0 | Left Lingual |
|  |  |  | 7.2 | -30 | -72 | -12 | Left Fusiform |
|  |  |  | 6.45 | -6 | -69 | 0 | Left Lingual |
|  |  |  | 5.65 | 6 | -72 | 9 | Right Calcarine |
|  |  |  | 5.64 | -9 | -75 | 12 | Left Calcarine |
|  |  |  | 5.46 | 9 | -81 | 6 | Right Calcarine |
|  |  |  | 5.11 | -24 | -60 | -3 | Left Lingual |
|  |  |  | 4.58 | 18 | -63 | 9 | Right Calcarine |
|  |  |  | 4.45 | 12 | -66 | 18 | Right Calcarine |
|  |  |  | 4.42 | 9 | -78 | -3 | Right Lingual |
|  | 80 | 0.001 | 7.11 | 9 | -45 | 45 | Right Precuneus |
|  |  |  | 4.37 | -6 | -54 | 48 | Left Precuneus |
|  | 382 | 0 | 7.03 | -42 | -66 | 0 | Left Middle Occipital |
|  |  |  | 6.81 | -27 | -84 | 18 | Left Middle Occipital |
|  |  |  | 6.49 | -42 | -63 | 12 | Left Middle Temporal |
|  |  |  | 4.98 | -27 | -72 | 27 | Left Middle Occipital |
|  |  |  | 4.86 | -33 | -90 | 12 | Left Middle Occipital |
|  |  |  | 4.64 | -48 | -75 | 6 | Left Middle Occipital |
|  |  |  | 4.51 | -36 | -81 | 12 | Left Middle Occipital |
|  | 132 | 0 | 6.82 | -30 | -51 | 48 | Left Inferior Parietal |
|  | 510 | 0 | 6.34 | 45 | -63 | 6 | Right Middle Temporal |
|  |  |  | 5.96 | 48 | -42 | 15 | Right Superior Temporal |
|  |  |  | 5.35 | 42 | -72 | 9 | Right Middle Temporal |
|  |  |  | 5.24 | 30 | -75 | 36 | Right Middle Occipital |
|  |  |  | 5.21 | 57 | -39 | 12 | Right Middle Temporal |
|  |  |  | 5.04 | 15 | -69 | 36 | Right Cuneus |
|  |  |  | 4.81 | 60 | -45 | 3 | Right Middle Temporal |
|  |  |  | 4.73 | 36 | -81 | 30 | Right Middle Occipital |
|  |  |  | 4.63 | 39 | -75 | 18 | Right Middle Occipital |
|  |  |  | 4.57 | 18 | -81 | 45 | Right Cuneus |
|  | 110 | 0 | 5.77 | 42 | 12 | 30 | Right Inferior Frontal Operculum |
|  |  |  | 5.59 | 51 | 12 | 36 | Right Precentral |
|  |  |  | 4.97 | 45 | 6 | 36 | Right Precentral |
|  | 63 | 0.003 | 5.5 | 36 | -39 | 48 | Right Inferior Parietal |
|  |  |  | 3.81 | 45 | -30 | 42 | Right Supramarginal |
|  | 62 | 0.004 | 5.35 | 39 | 3 | 54 | Right Middle Frontal |
|  |  |  | 4.41 | 30 | -3 | 54 | Right Middle Frontal |
|  | 48 | 0.013 | 5.01 | -48 | 6 | 30 | Left Precentral |
|  | 53 | 0.008 | 4.95 | 45 | 18 | 9 | Right Frontal inferior Operculum |
|  |  |  | 4.43 | 36 | 18 | -3 | Right Insula |
|  |  |  | 3.94 | 36 | 27 | -3 | Right Insula |
| Controls: Redirect > No-Step | | | | | | | |
|  | 8773 | 0 | 10.79 | -33 | -45 | 51 | Left Inferior Parietal |
|  |  |  | 10.68 | -15 | -75 | 39 | Left Cuneus |
|  |  |  | 10.18 | 18 | -69 | 33 | Right Cuneus |
|  |  |  | 9.9 | -9 | -90 | 15 | Left Cuneus |
|  |  |  | 9.73 | 3 | -75 | 12 | Right Calcarine |
|  |  |  | 9.56 | 27 | -81 | 18 | Right Middle Occipital |
|  |  |  | 9.25 | -30 | -57 | 54 | Left Inferior Parietal |
|  |  |  | 9.2 | 63 | -45 | 24 | Right Superior Temporal |
|  |  |  | 8.99 | 27 | -63 | 51 | Right Superior Parietal |
|  |  |  | 8.9 | -27 | -75 | 24 | Left Middle Occipital |
|  | 2357 | 0 | 9.39 | -54 | 3 | 42 | Left Precentral |
|  |  |  | 9.08 | 24 | 3 | 69 | Right Superior Frontal |
|  |  |  | 8.78 | 30 | 3 | 63 | Right Superior Frontal |
|  |  |  | 8.46 | -9 | 9 | 54 | Left Supplementary motor area |
|  |  |  | 8.38 | -21 | 0 | 54 | Left Superior Frontal |
|  |  |  | 7.83 | 48 | 15 | 30 | Right Inferior frontal operculum |
|  |  |  | 7.1 | -24 | -6 | 63 | Left Superior Frontal |
|  |  |  | 6.96 | 30 | -3 | 48 | Right Precentral |
|  |  |  | 6.89 | -45 | 3 | 36 | Left Precentral |
|  |  |  | 6.88 | 54 | 9 | 33 | Right Precentral |
|  | 199 | 0 | 6.15 | -30 | 21 | 3 | Left Insula |
|  |  |  | 5.76 | -33 | 18 | 12 | Left Insula |
|  |  |  | 5.32 | -33 | 21 | -6 | Left Insula |
|  |  |  | 5.28 | -39 | 15 | -6 | Left Insula |
|  |  |  | 3.61 | -18 | 27 | -3 | Left Insula |
| Patients > Controls: No-Step > Fixation | | | | | | | |
|  | 180 | 0.000 | 5.07 | 24 | -63 | -18 | Right Cerebellum 6 |
|  |  |  | 4.68 | 24 | -78 | -18 | Right Cerebellum 6 |
|  |  |  | 4.49 | 30 | -57 | -15 | Right Fusiform |
|  |  |  | 4.18 | 24 | -60 | -27 | Right Cerebellum 6 |
|  |  |  | 4.05 | 21 | -57 | -3 | Right Lingual |
|  |  |  | 4.05 | 36 | -72 | -18 | Right Fusiform |
|  |  |  | 3.44 | 27 | -45 | -15 | Right Fusiform |
|  |  |  | 3.42 | 27 | -51 | -6 | Right Fusiform |
|  | 63 | 0.015 | 4.75 | 24 | -63 | 54 | Right Superior Parietal |
|  |  |  | 3.92 | 27 | -72 | 51 | Right Superior Parietal |
|  |  |  | 3.5 | 30 | -63 | 42 | Right Superior Occipital |
|  | 55 | 0.027 | 4.61 | -15 | -54 | -27 | Left Cerebellum 6 |
|  |  |  | 4.12 | -27 | -54 | -27 | Left Cerebellum 6 |
|  |  |  | 3.93 | -21 | -45 | -15 | Left Fusiform |
| Control > Patients: Redirect > No-Step | | | | | | | |
|  | 90 | 0.001 | 4.76 | -24 | -87 | 3 | Left Middle Occipital |
|  |  |  | 4.08 | -36 | -81 | 3 | Left Middle Occipital |
|  |  |  | 3.82 | -36 | -81 | -9 | Left Inferior Occipital |
|  | 54 | 0.015 | 4.34 | 9 | -51 | 60 | Right Precuneus |
|  |  |  | 4.26 | 12 | -60 | 57 | Right Precuneus |
|  |  |  | 3.91 | 24 | -54 | 60 | Right Superior Parietal |

| Table S2. Control DCM parameters | |  |  |  |
| --- | --- | --- | --- | --- |
| Connection type | Connection | | BMA Probability | BMA parameter |
| Mean Effective Connectivity | From FEF to FEF | | 0.00 | 0.00 |
| Mean Effective Connectivity | From FEF to SEF | | 1.00 | -0.25 |
| Mean Effective Connectivity | From FEF to CD | | 0.59 | -0.02 |
| Mean Effective Connectivity | From FEF to Thal | | 1.00 | -0.11 |
| Mean Effective Connectivity | From FEF to SC | | 1.00 | -0.21 |
| Mean Effective Connectivity | From SEF to FEF | | 1.00 | 0.12 |
| Mean Effective Connectivity | From SEF to SEF | | 1.00 | -0.27 |
| Mean Effective Connectivity | From SEF to CD | | 0.00 | 0.00 |
| Mean Effective Connectivity | From SEF to Thal | | 0.00 | 0.00 |
| Mean Effective Connectivity | From SEF to SC | | 0.00 | 0.00 |
| Mean Effective Connectivity | From SEF to IFC | | 0.00 | 0.00 |
| Mean Effective Connectivity | From CD to CD | | 1.00 | -0.71 |
| Mean Effective Connectivity | From CD to Thal | | 1.00 | 0.20 |
| Mean Effective Connectivity | From CD to SC | | 1.00 | 0.31 |
| Mean Effective Connectivity | From Thal to FEF | | 1.00 | 0.29 |
| Mean Effective Connectivity | From Thal to SEF | | 1.00 | 0.38 |
| Mean Effective Connectivity | From Thal to Thal | | 1.00 | -0.28 |
| Mean Effective Connectivity | From Thal to IFC | | 0.72 | 0.07 |
| Mean Effective Connectivity | From SC to CD | | 0.67 | 0.03 |
| Mean Effective Connectivity | From SC to Thal | | 1.00 | 0.13 |
| Mean Effective Connectivity | From SC to SC | | 1.00 | -0.53 |
| Mean Effective Connectivity | From IFC to SEF | | 1.00 | 0.10 |
| Mean Effective Connectivity | From IFC to CD | | 0.00 | 0.00 |
| Mean Effective Connectivity | From IFC to Thal | | 0.00 | 0.00 |
| Mean Effective Connectivity | From IFC to IFC | | 0.90 | -0.09 |
| Connection type | Connection | | BMA Probability | BMA parameter |
| Modulation due to Redirect | From FEF to FEF | | 1.00 | -1.75 |
| Modulation due to Redirect | From FEF to SEF | | 1.00 | 0.88 |
| Modulation due to Redirect | From FEF to CD | | 0.76 | 0.24 |
| Modulation due to Redirect | From FEF to Thal | | 0.00 | 0.00 |
| Modulation due to Redirect | From FEF to SC | | 0.00 | 0.00 |
| Modulation due to Redirect | From SEF to FEF | | 0.86 | 0.40 |
| Modulation due to Redirect | From SEF to SEF | | 1.00 | -1.91 |
| Modulation due to Redirect | From SEF to CD | | 0.65 | 0.20 |
| Modulation due to Redirect | From SEF to Thal | | 0.00 | 0.00 |
| Modulation due to Redirect | From SEF to SC | | 0.00 | 0.00 |
| Modulation due to Redirect | From SEF to IFC | | 1.00 | 1.20 |
| Modulation due to Redirect | From CD to CD | | 0.00 | 0.00 |
| Modulation due to Redirect | From CD to Thal | | 0.00 | 0.00 |
| Modulation due to Redirect | From CD to SC | | 1.00 | 1.49 |
| Modulation due to Redirect | From Thal to FEF | | 0.00 | 0.00 |
| Modulation due to Redirect | From Thal to SEF | | 0.00 | 0.00 |
| Modulation due to Redirect | From Thal to Thal | | 1.00 | -1.33 |
| Modulation due to Redirect | From Thal to IFC | | 0.00 | 0.00 |
| Modulation due to Redirect | From SC to CD | | 0.00 | 0.00 |
| Modulation due to Redirect | From SC to Thal | | 1.00 | 0.89 |
| Modulation due to Redirect | From SC to SC | | 1.00 | -1.65 |
| Modulation due to Redirect | From IFC to SEF | | 0.00 | 0.00 |
| Modulation due to Redirect | From IFC to CD | | 0.00 | 0.00 |
| Modulation due to Redirect | From IFC to Thal | | 0.63 | -0.18 |
| Modulation due to Redirect | From IFC to IFC | | 1.00 | -1.27 |
| Note: The PEB and BMA model was not optimized over the C matrix, so C parameters are not estimated in the BMA. | | | | |

| Table S3. Patient DCM parameters | | | |
| --- | --- | --- | --- |
| Connection type | Connection | BMA Probability | BMA parameter |
| Mean Effective Connectivity | From FEF to FEF | 1.00 | -0.21 |
| Mean Effective Connectivity | From FEF to SEF | 0.00 | 0.00 |
| Mean Effective Connectivity | From FEF to CD | 0.00 | 0.00 |
| Mean Effective Connectivity | From FEF to Thal | 1.00 | -0.16 |
| Mean Effective Connectivity | From FEF to SC | 0.99 | -0.08 |
| Mean Effective Connectivity | From SEF to FEF | 1.00 | 0.15 |
| Mean Effective Connectivity | From SEF to SEF | 1.00 | -0.64 |
| Mean Effective Connectivity | From SEF to CD | 1.00 | -0.10 |
| Mean Effective Connectivity | From SEF to Thal | 0.00 | 0.00 |
| Mean Effective Connectivity | From SEF to SC | 0.00 | 0.00 |
| Mean Effective Connectivity | From SEF to IFC | 1.00 | 0.12 |
| Mean Effective Connectivity | From CD to CD | 1.00 | -0.80 |
| Mean Effective Connectivity | From CD to Thal | 1.00 | 0.19 |
| Mean Effective Connectivity | From CD to SC | 1.00 | 0.23 |
| Mean Effective Connectivity | From Thal to FEF | 1.00 | 0.41 |
| Mean Effective Connectivity | From Thal to SEF | 1.00 | 0.24 |
| Mean Effective Connectivity | From Thal to Thal | 1.00 | -0.55 |
| Mean Effective Connectivity | From Thal to IFC | 1.00 | 0.37 |
| Mean Effective Connectivity | From SC to CD | 1.00 | 0.14 |
| Mean Effective Connectivity | From SC to Thal | 0.00 | 0.00 |
| Mean Effective Connectivity | From SC to SC | 1.00 | -0.28 |
| Mean Effective Connectivity | From IFC to SEF | 1.00 | -0.20 |
| Mean Effective Connectivity | From IFC to CD | 0.00 | 0.00 |
| Mean Effective Connectivity | From IFC to Thal | 1.00 | 0.17 |
| Mean Effective Connectivity | From IFC to IFC | 0.69 | 0.05 |
| Connection type | Connection | BMA Probability | BMA parameter |
| Modulation due to Redirect | From FEF to FEF | 0.99 | -0.69 |
| Modulation due to Redirect | From FEF to SEF | 0.97 | -0.44 |
| Modulation due to Redirect | From FEF to CD | 0.00 | 0.00 |
| Modulation due to Redirect | From FEF to Thal | 0.00 | 0.00 |
| Modulation due to Redirect | From FEF to SC | 0.00 | 0.00 |
| Modulation due to Redirect | From SEF to FEF | 0.00 | 0.00 |
| Modulation due to Redirect | From SEF to SEF | 1.00 | -1.88 |
| Modulation due to Redirect | From SEF to CD | 0.00 | 0.00 |
| Modulation due to Redirect | From SEF to Thal | 0.00 | 0.00 |
| Modulation due to Redirect | From SEF to SC | 0.00 | 0.00 |
| Modulation due to Redirect | From SEF to IFC | 0.00 | 0.00 |
| Modulation due to Redirect | From CD to CD | 1.00 | -1.35 |
| Modulation due to Redirect | From CD to Thal | 0.00 | 0.00 |
| Modulation due to Redirect | From CD to SC | 0.97 | 0.65 |
| Modulation due to Redirect | From Thal to FEF | 1.00 | 1.26 |
| Modulation due to Redirect | From Thal to SEF | 0.00 | 0.00 |
| Modulation due to Redirect | From Thal to Thal | 1.00 | -2.06 |
| Modulation due to Redirect | From Thal to IFC | 0.00 | 0.00 |
| Modulation due to Redirect | From SC to CD | 0.00 | 0.00 |
| Modulation due to Redirect | From SC to Thal | 0.00 | 0.00 |
| Modulation due to Redirect | From SC to SC | 0.76 | -0.50 |
| Modulation due to Redirect | From IFC to SEF | 0.99 | 0.56 |
| Modulation due to Redirect | From IFC to CD | 0.88 | 0.35 |
| Modulation due to Redirect | From IFC to Thal | 0.00 | 0.00 |
| Modulation due to Redirect | From IFC to IFC | 1.00 | -1.88 |
| Note: The PEB and BMA model was not optimized over the C matrix, so C parameters are not estimated in the BMA. | | | |

| Table S4. Group DCM analysis. | | | |
| --- | --- | --- | --- |
| Connection Type | Connection | BMA Probability | BMA parameter |
| Mean Effective Connectivity | From FEF to FEF | 0.00 | 0.00 |
| Mean Effective Connectivity | From FEF to SEF | 1.00 | -0.12 |
| Mean Effective Connectivity | From FEF to CD | 0.00 | 0.00 |
| Mean Effective Connectivity | From FEF to Thal | 1.00 | -0.10 |
| Mean Effective Connectivity | From FEF to SC | 1.00 | -0.17 |
| Mean Effective Connectivity | From SEF to FEF | 1.00 | 0.13 |
| Mean Effective Connectivity | From SEF to SEF | 1.00 | -0.31 |
| Mean Effective Connectivity | From SEF to CD | 0.00 | 0.00 |
| Mean Effective Connectivity | From SEF to Thal | 0.00 | 0.00 |
| Mean Effective Connectivity | From SEF to SC | 0.00 | 0.00 |
| Mean Effective Connectivity | From SEF to IFC | 0.00 | 0.00 |
| Mean Effective Connectivity | From CD to CD | 1.00 | -0.73 |
| Mean Effective Connectivity | From CD to Thal | 1.00 | 0.20 |
| Mean Effective Connectivity | From CD to SC | 1.00 | 0.28 |
| Mean Effective Connectivity | From Thal to FEF | 1.00 | 0.35 |
| Mean Effective Connectivity | From Thal to SEF | 1.00 | 0.33 |
| Mean Effective Connectivity | From Thal to Thal | 1.00 | -0.37 |
| Mean Effective Connectivity | From Thal to IFC | 1.00 | 0.24 |
| Mean Effective Connectivity | From SC to CD | 1.00 | 0.08 |
| Mean Effective Connectivity | From SC to Thal | 1.00 | 0.09 |
| Mean Effective Connectivity | From SC to SC | 1.00 | -0.36 |
| Mean Effective Connectivity | From IFC to SEF | 0.00 | 0.00 |
| Mean Effective Connectivity | From IFC to CD | 1.00 | -0.05 |
| Mean Effective Connectivity | From IFC to Thal | 0.00 | 0.00 |
| Mean Effective Connectivity | From IFC to IFC | 0.00 | 0.00 |
| Connection Type | Connection | BMA Probability | BMA parameter |
| Mean Modulation | From FEF to FEF | 1.00 | -1.25 |
| Mean Modulation | From FEF to SEF | 1.00 | 0.30 |
| Mean Modulation | From FEF to CD | 0.00 | 0.00 |
| Mean Modulation | From FEF to Thal | 0.00 | 0.00 |
| Mean Modulation | From FEF to SC | 0.00 | 0.00 |
| Mean Modulation | From SEF to FEF | 0.00 | 0.00 |
| Mean Modulation | From SEF to SEF | 1.00 | -1.81 |
| Mean Modulation | From SEF to CD | 0.00 | 0.00 |
| Mean Modulation | From SEF to Thal | 0.00 | 0.00 |
| Mean Modulation | From SEF to SC | 0.00 | 0.00 |
| Mean Modulation | From SEF to IFC | 1.00 | 0.72 |
| Mean Modulation | From CD to CD | 1.00 | -0.73 |
| Mean Modulation | From CD to Thal | 0.00 | 0.00 |
| Mean Modulation | From CD to SC | 1.00 | 1.18 |
| Mean Modulation | From Thal to FEF | 1.00 | 0.69 |
| Mean Modulation | From Thal to SEF | 0.00 | 0.00 |
| Mean Modulation | From Thal to Thal | 1.00 | -1.37 |
| Mean Modulation | From Thal to IFC | 0.00 | 0.00 |
| Mean Modulation | From SC to CD | 0.00 | 0.00 |
| Mean Modulation | From SC to Thal | 1.00 | 0.48 |
| Mean Modulation | From SC to SC | 1.00 | -1.25 |
| Mean Modulation | From IFC to SEF | 0.00 | 0.00 |
| Mean Modulation | From IFC to CD | 1.00 | 0.26 |
| Mean Modulation | From IFC to Thal | 1.00 | -0.24 |
| Mean Modulation | From IFC to IFC | 1.00 | -1.34 |
| Connection Type | Connection | BMA Probability | BMA parameter |
| Group Differences in Mean Connectivity | From FEF to FEF | 1.00 | -0.07 |
| Group Differences in Mean Connectivity | From FEF to SEF | 1.00 | 0.13 |
| Group Differences in Mean Connectivity | From FEF to CD | 0.00 | 0.00 |
| Group Differences in Mean Connectivity | From FEF to Thal | 0.00 | 0.00 |
| Group Differences in Mean Connectivity | From FEF to SC | 1.00 | 0.05 |
| Group Differences in Mean Connectivity | From SEF to FEF | 0.00 | 0.00 |
| Group Differences in Mean Connectivity | From SEF to SEF | 1.00 | -0.08 |
| Group Differences in Mean Connectivity | From SEF to CD | 1.00 | -0.04 |
| Group Differences in Mean Connectivity | From SEF to Thal | 0.00 | 0.00 |
| Group Differences in Mean Connectivity | From SEF to SC | 0.00 | 0.00 |
| Group Differences in Mean Connectivity | From SEF to IFC | 0.00 | 0.00 |
| Group Differences in Mean Connectivity | From CD to CD | 0.00 | 0.00 |
| Group Differences in Mean Connectivity | From CD to Thal | 0.00 | 0.00 |
| Group Differences in Mean Connectivity | From CD to SC | 0.00 | 0.00 |
| Group Differences in Mean Connectivity | From Thal to FEF | 0.53 | 0.03 |
| Group Differences in Mean Connectivity | From Thal to SEF | 0.69 | -0.05 |
| Group Differences in Mean Connectivity | From Thal to Thal | 1.00 | -0.10 |
| Group Differences in Mean Connectivity | From Thal to IFC | 1.00 | 0.14 |
| Group Differences in Mean Connectivity | From SC to CD | 0.61 | 0.03 |
| Group Differences in Mean Connectivity | From SC to Thal | 0.72 | -0.04 |
| Group Differences in Mean Connectivity | From SC to SC | 1.00 | 0.19 |
| Group Differences in Mean Connectivity | From IFC to SEF | 1.00 | -0.12 |
| Group Differences in Mean Connectivity | From IFC to CD | 0.00 | 0.00 |
| Group Differences in Mean Connectivity | From IFC to Thal | 1.00 | 0.04 |
| Group Differences in Mean Connectivity | From IFC to IFC | 1.00 | 0.12 |
| Connection Type | Connection | BMA Probability | BMA parameter |
| Group Differences in Modulation | From FEF to FEF | 1.00 | 0.60 |
| Group Differences in Modulation | From FEF to SEF | 1.00 | -0.51 |
| Group Differences in Modulation | From FEF to CD | 0.00 | 0.00 |
| Group Differences in Modulation | From FEF to Thal | 0.00 | 0.00 |
| Group Differences in Modulation | From FEF to SC | 0.00 | 0.00 |
| Group Differences in Modulation | From SEF to FEF | 0.00 | 0.00 |
| Group Differences in Modulation | From SEF to SEF | 0.00 | 0.00 |
| Group Differences in Modulation | From SEF to CD | 0.00 | 0.00 |
| Group Differences in Modulation | From SEF to Thal | 0.00 | 0.00 |
| Group Differences in Modulation | From SEF to SC | 0.00 | 0.00 |
| Group Differences in Modulation | From SEF to IFC | 1.00 | -0.36 |
| Group Differences in Modulation | From CD to CD | 1.00 | -0.66 |
| Group Differences in Modulation | From CD to Thal | 0.00 | 0.00 |
| Group Differences in Modulation | From CD to SC | 0.72 | -0.30 |
| Group Differences in Modulation | From Thal to FEF | 0.63 | 0.25 |
| Group Differences in Modulation | From Thal to SEF | 0.00 | 0.00 |
| Group Differences in Modulation | From Thal to Thal | 0.00 | 0.00 |
| Group Differences in Modulation | From Thal to IFC | 0.64 | -0.28 |
| Group Differences in Modulation | From SC to CD | 0.00 | 0.00 |
| Group Differences in Modulation | From SC to Thal | 1.00 | -0.31 |
| Group Differences in Modulation | From SC to SC | 0.78 | 0.42 |
| Group Differences in Modulation | From IFC to SEF | 0.00 | 0.00 |
| Group Differences in Modulation | From IFC to CD | 0.00 | 0.00 |
| Group Differences in Modulation | From IFC to Thal | 0.00 | 0.00 |
| Group Differences in Modulation | From IFC to IFC | 0.00 | 0.00 |

| Table S5. Supplemental Control DCM Parameters with Modulation on Compensated Trials. | | | |
| --- | --- | --- | --- |
| Connection type | Connection | BMA Probability | BMA parameter |
| Mean Effective Connectivity | From FEF to FEF | 0.64 | 0.05 |
| Mean Effective Connectivity | From FEF to SEF | 1.00 | -0.33 |
| Mean Effective Connectivity | From FEF to CD | 0.87 | 0.05 |
| Mean Effective Connectivity | From FEF to Thal | 0.00 | 0.00 |
| Mean Effective Connectivity | From FEF to SC | 1.00 | -0.19 |
| Mean Effective Connectivity | From SEF to FEF | 1.00 | 0.10 |
| Mean Effective Connectivity | From SEF to SEF | 1.00 | -0.69 |
| Mean Effective Connectivity | From SEF to CD | 0.00 | 0.00 |
| Mean Effective Connectivity | From SEF to Thal | 0.00 | 0.00 |
| Mean Effective Connectivity | From SEF to SC | 0.00 | 0.00 |
| Mean Effective Connectivity | From SEF to IFC | 0.73 | 0.06 |
| Mean Effective Connectivity | From CD to CD | 1.00 | -0.79 |
| Mean Effective Connectivity | From CD to Thal | 1.00 | 0.21 |
| Mean Effective Connectivity | From CD to SC | 1.00 | 0.42 |
| Mean Effective Connectivity | From Thal to FEF | 1.00 | 0.57 |
| Mean Effective Connectivity | From Thal to SEF | 1.00 | 0.60 |
| Mean Effective Connectivity | From Thal to Thal | 1.00 | -0.60 |
| Mean Effective Connectivity | From Thal to IFC | 1.00 | 0.59 |
| Mean Effective Connectivity | From SC to CD | 1.00 | -0.12 |
| Mean Effective Connectivity | From SC to Thal | 0.00 | 0.00 |
| Mean Effective Connectivity | From SC to SC | 1.00 | -0.51 |
| Mean Effective Connectivity | From IFC to SEF | 0.00 | 0.00 |
| Mean Effective Connectivity | From IFC to CD | 0.00 | 0.00 |
| Mean Effective Connectivity | From IFC to Thal | 1.00 | -0.07 |
| Mean Effective Connectivity | From IFC to IFC | 0.73 | 0.07 |
| Connection type | Connection | BMA Probability | BMA parameter |
| Modulation due to Redirect | From FEF to FEF | 1.00 | -0.72 |
| Modulation due to Redirect | From FEF to SEF | 1.00 | 0.68 |
| Modulation due to Redirect | From FEF to CD | 0.00 | 0.00 |
| Modulation due to Redirect | From FEF to Thal | 0.72 | -0.22 |
| Modulation due to Redirect | From FEF to SC | 0.79 | -0.31 |
| Modulation due to Redirect | From SEF to FEF | 1.00 | 0.54 |
| Modulation due to Redirect | From SEF to SEF | 1.00 | -1.79 |
| Modulation due to Redirect | From SEF to CD | 0.00 | 0.00 |
| Modulation due to Redirect | From SEF to Thal | 0.00 | 0.00 |
| Modulation due to Redirect | From SEF to SC | 1.00 | 0.50 |
| Modulation due to Redirect | From SEF to IFC | 1.00 | 0.77 |
| Modulation due to Redirect | From CD to CD | 1.00 | -3.04 |
| Modulation due to Redirect | From CD to Thal | 0.00 | 0.00 |
| Modulation due to Redirect | From CD to SC | 0.00 | 0.00 |
| Modulation due to Redirect | From Thal to FEF | 0.80 | -0.36 |
| Modulation due to Redirect | From Thal to SEF | 1.00 | -0.95 |
| Modulation due to Redirect | From Thal to Thal | 1.00 | -3.82 |
| Modulation due to Redirect | From Thal to IFC | 0.00 | 0.00 |
| Modulation due to Redirect | From SC to CD | 0.00 | 0.00 |
| Modulation due to Redirect | From SC to Thal | 0.00 | 0.00 |
| Modulation due to Redirect | From SC to SC | 1.00 | -1.17 |
| Modulation due to Redirect | From IFC to SEF | 1.00 | -0.62 |
| Modulation due to Redirect | From IFC to CD | 0.00 | 0.00 |
| Modulation due to Redirect | From IFC to Thal | 0.00 | 0.00 |
| Modulation due to Redirect | From IFC to IFC | 0.78 | -0.53 |
| Note: The PEB and BMA model was not optimized over the C matrix, so C parameters are not estimated in the BMA. | | | |

| Table S6. Supplemental Patient DCM Parameters with Modulation on Compensated Trials. | | | |
| --- | --- | --- | --- |
| Connection type | Connection | BMA Probability | BMA parameter |
| Mean Effective Connectivity | From FEF to FEF | 0.95 | -0.10 |
| Mean Effective Connectivity | From FEF to SEF | 0.00 | 0.00 |
| Mean Effective Connectivity | From FEF to CD | 1.00 | -0.09 |
| Mean Effective Connectivity | From FEF to Thal | 1.00 | -0.16 |
| Mean Effective Connectivity | From FEF to SC | 1.00 | -0.08 |
| Mean Effective Connectivity | From SEF to FEF | 1.00 | 0.15 |
| Mean Effective Connectivity | From SEF to SEF | 1.00 | -0.18 |
| Mean Effective Connectivity | From SEF to CD | 0.69 | 0.04 |
| Mean Effective Connectivity | From SEF to Thal | 0.82 | 0.06 |
| Mean Effective Connectivity | From SEF to SC | 0.81 | 0.05 |
| Mean Effective Connectivity | From SEF to IFC | 1.00 | 0.17 |
| Mean Effective Connectivity | From CD to CD | 1.00 | -0.59 |
| Mean Effective Connectivity | From CD to Thal | 1.00 | -0.18 |
| Mean Effective Connectivity | From CD to SC | 1.00 | -0.23 |
| Mean Effective Connectivity | From Thal to FEF | 1.00 | 0.52 |
| Mean Effective Connectivity | From Thal to SEF | 1.00 | 0.15 |
| Mean Effective Connectivity | From Thal to Thal | 1.00 | -0.81 |
| Mean Effective Connectivity | From Thal to IFC | 0.93 | 0.12 |
| Mean Effective Connectivity | From SC to CD | 0.00 | 0.00 |
| Mean Effective Connectivity | From SC to Thal | 1.00 | 0.14 |
| Mean Effective Connectivity | From SC to SC | 1.00 | -0.57 |
| Mean Effective Connectivity | From IFC to SEF | 0.00 | 0.00 |
| Mean Effective Connectivity | From IFC to CD | 0.83 | 0.05 |
| Mean Effective Connectivity | From IFC to Thal | 1.00 | 0.09 |
| Mean Effective Connectivity | From IFC to IFC | 0.92 | 0.12 |
| Connection type | Connection | BMA Probability | BMA parameter |
| Modulation due to Redirect | From FEF to FEF | 1.00 | -0.82 |
| Modulation due to Redirect | From FEF to SEF | 0.00 | 0.00 |
| Modulation due to Redirect | From FEF to CD | 0.00 | 0.00 |
| Modulation due to Redirect | From FEF to Thal | 0.00 | 0.00 |
| Modulation due to Redirect | From FEF to SC | 0.00 | 0.00 |
| Modulation due to Redirect | From SEF to FEF | 0.00 | 0.00 |
| Modulation due to Redirect | From SEF to SEF | 1.00 | -1.56 |
| Modulation due to Redirect | From SEF to CD | 0.00 | 0.00 |
| Modulation due to Redirect | From SEF to Thal | 0.00 | 0.00 |
| Modulation due to Redirect | From SEF to SC | 0.00 | 0.00 |
| Modulation due to Redirect | From SEF to IFC | 0.00 | 0.00 |
| Modulation due to Redirect | From CD to CD | 1.00 | -2.33 |
| Modulation due to Redirect | From CD to Thal | 0.00 | 0.00 |
| Modulation due to Redirect | From CD to SC | 1.00 | 0.96 |
| Modulation due to Redirect | From Thal to FEF | 0.00 | 0.00 |
| Modulation due to Redirect | From Thal to SEF | 0.00 | 0.00 |
| Modulation due to Redirect | From Thal to Thal | 1.00 | -2.84 |
| Modulation due to Redirect | From Thal to IFC | 1.00 | 1.04 |
| Modulation due to Redirect | From SC to CD | 0.00 | 0.00 |
| Modulation due to Redirect | From SC to Thal | 0.83 | -0.41 |
| Modulation due to Redirect | From SC to SC | 1.00 | -1.83 |
| Modulation due to Redirect | From IFC to SEF | 0.00 | 0.00 |
| Modulation due to Redirect | From IFC to CD | 0.00 | 0.00 |
| Modulation due to Redirect | From IFC to Thal | 0.00 | 0.00 |
| Modulation due to Redirect | From IFC to IFC | 1.00 | -1.04 |
| Note: The PEB and BMA model was not optimized over the C matrix, so C parameters are not estimated in the BMA. | | | |

| Table S7. Supplemental Group DCM analysis with Modulation on Compensated Trials. | | | |
| --- | --- | --- | --- |
| Connection Type | Connection | BMA Probability | BMA parameter |
| Mean Effective Connectivity | From FEF to FEF | 1.00 | 0.08 |
| Mean Effective Connectivity | From FEF to SEF | 1.00 | -0.20 |
| Mean Effective Connectivity | From FEF to CD | 0.00 | 0.00 |
| Mean Effective Connectivity | From FEF to Thal | 1.00 | -0.08 |
| Mean Effective Connectivity | From FEF to SC | 1.00 | -0.16 |
| Mean Effective Connectivity | From SEF to FEF | 1.00 | 0.13 |
| Mean Effective Connectivity | From SEF to SEF | 1.00 | -0.40 |
| Mean Effective Connectivity | From SEF to CD | 0.00 | 0.00 |
| Mean Effective Connectivity | From SEF to Thal | 0.00 | 0.00 |
| Mean Effective Connectivity | From SEF to SC | 1.00 | 0.05 |
| Mean Effective Connectivity | From SEF to IFC | 1.00 | 0.11 |
| Mean Effective Connectivity | From CD to CD | 1.00 | -0.66 |
| Mean Effective Connectivity | From CD to Thal | 0.00 | 0.00 |
| Mean Effective Connectivity | From CD to SC | 1.00 | 0.09 |
| Mean Effective Connectivity | From Thal to FEF | 1.00 | 0.55 |
| Mean Effective Connectivity | From Thal to SEF | 1.00 | 0.34 |
| Mean Effective Connectivity | From Thal to Thal | 1.00 | -0.69 |
| Mean Effective Connectivity | From Thal to IFC | 1.00 | 0.28 |
| Mean Effective Connectivity | From SC to CD | 1.00 | -0.06 |
| Mean Effective Connectivity | From SC to Thal | 1.00 | 0.07 |
| Mean Effective Connectivity | From SC to SC | 1.00 | -0.52 |
| Mean Effective Connectivity | From IFC to SEF | 0.00 | 0.00 |
| Mean Effective Connectivity | From IFC to CD | 0.50 | 0.02 |
| Mean Effective Connectivity | From IFC to Thal | 0.00 | 0.00 |
| Mean Effective Connectivity | From IFC to IFC | 1.00 | 0.24 |
| Connection Type | Connection | BMA Probability | BMA parameter |
| Mean Modulation | From FEF to FEF | 1.00 | -0.75 |
| Mean Modulation | From FEF to SEF | 0.00 | 0.00 |
| Mean Modulation | From FEF to CD | 0.00 | 0.00 |
| Mean Modulation | From FEF to Thal | 0.61 | -0.14 |
| Mean Modulation | From FEF to SC | 0.00 | 0.00 |
| Mean Modulation | From SEF to FEF | 1.00 | 0.41 |
| Mean Modulation | From SEF to SEF | 1.00 | -1.70 |
| Mean Modulation | From SEF to CD | 0.00 | 0.00 |
| Mean Modulation | From SEF to Thal | 0.00 | 0.00 |
| Mean Modulation | From SEF to SC | 0.00 | 0.00 |
| Mean Modulation | From SEF to IFC | 0.00 | 0.00 |
| Mean Modulation | From CD to CD | 1.00 | -2.64 |
| Mean Modulation | From CD to Thal | 0.00 | 0.00 |
| Mean Modulation | From CD to SC | 1.00 | 0.45 |
| Mean Modulation | From Thal to FEF | 1.00 | -0.48 |
| Mean Modulation | From Thal to SEF | 1.00 | -0.65 |
| Mean Modulation | From Thal to Thal | 1.00 | -2.97 |
| Mean Modulation | From Thal to IFC | 1.00 | 0.52 |
| Mean Modulation | From SC to CD | 0.00 | 0.00 |
| Mean Modulation | From SC to Thal | 0.00 | 0.00 |
| Mean Modulation | From SC to SC | 1.00 | -1.48 |
| Mean Modulation | From IFC to SEF | 0.00 | 0.00 |
| Mean Modulation | From IFC to CD | 0.00 | 0.00 |
| Mean Modulation | From IFC to Thal | 0.00 | 0.00 |
| Mean Modulation | From IFC to IFC | 1.00 | -1.22 |
| Connection Type | Connection | BMA Probability | BMA parameter |
| Group Differences in Mean Connectivity | From FEF to FEF | 1.00 | -0.07 |
| Group Differences in Mean Connectivity | From FEF to SEF | 1.00 | 0.13 |
| Group Differences in Mean Connectivity | From FEF to CD | 1.00 | -0.06 |
| Group Differences in Mean Connectivity | From FEF to Thal | 1.00 | -0.05 |
| Group Differences in Mean Connectivity | From FEF to SC | 1.00 | 0.05 |
| Group Differences in Mean Connectivity | From SEF to FEF | 0.00 | 0.00 |
| Group Differences in Mean Connectivity | From SEF to SEF | 1.00 | 0.25 |
| Group Differences in Mean Connectivity | From SEF to CD | 0.63 | 0.03 |
| Group Differences in Mean Connectivity | From SEF to Thal | 0.00 | 0.00 |
| Group Differences in Mean Connectivity | From SEF to SC | 0.00 | 0.00 |
| Group Differences in Mean Connectivity | From SEF to IFC | 0.57 | 0.03 |
| Group Differences in Mean Connectivity | From CD to CD | 1.00 | 0.17 |
| Group Differences in Mean Connectivity | From CD to Thal | 1.00 | -0.18 |
| Group Differences in Mean Connectivity | From CD to SC | 1.00 | -0.30 |
| Group Differences in Mean Connectivity | From Thal to FEF | 0.00 | 0.00 |
| Group Differences in Mean Connectivity | From Thal to SEF | 1.00 | -0.16 |
| Group Differences in Mean Connectivity | From Thal to Thal | 1.00 | -0.11 |
| Group Differences in Mean Connectivity | From Thal to IFC | 1.00 | -0.17 |
| Group Differences in Mean Connectivity | From SC to CD | 1.00 | 0.07 |
| Group Differences in Mean Connectivity | From SC to Thal | 1.00 | 0.06 |
| Group Differences in Mean Connectivity | From SC to SC | 0.00 | 0.00 |
| Group Differences in Mean Connectivity | From IFC to SEF | 0.00 | 0.00 |
| Group Differences in Mean Connectivity | From IFC to CD | 0.00 | 0.00 |
| Group Differences in Mean Connectivity | From IFC to Thal | 1.00 | 0.05 |
| Group Differences in Mean Connectivity | From IFC to IFC | 0.00 | 0.00 |
| Connection Type | Connection | BMA Probability | BMA parameter |
| Group Differences in Modulation | From FEF to FEF | 0.00 | 0.00 |
| Group Differences in Modulation | From FEF to SEF | 0.69 | -0.19 |
| Group Differences in Modulation | From FEF to CD | 0.00 | 0.00 |
| Group Differences in Modulation | From FEF to Thal | 0.00 | 0.00 |
| Group Differences in Modulation | From FEF to SC | 0.00 | 0.00 |
| Group Differences in Modulation | From SEF to FEF | 0.00 | 0.00 |
| Group Differences in Modulation | From SEF to SEF | 0.00 | 0.00 |
| Group Differences in Modulation | From SEF to CD | 0.00 | 0.00 |
| Group Differences in Modulation | From SEF to Thal | 0.00 | 0.00 |
| Group Differences in Modulation | From SEF to SC | 0.60 | -0.14 |
| Group Differences in Modulation | From SEF to IFC | 1.00 | -0.56 |
| Group Differences in Modulation | From CD to CD | 0.00 | 0.00 |
| Group Differences in Modulation | From CD to Thal | 0.00 | 0.00 |
| Group Differences in Modulation | From CD to SC | 1.00 | 0.42 |
| Group Differences in Modulation | From Thal to FEF | 0.00 | 0.00 |
| Group Differences in Modulation | From Thal to SEF | 0.00 | 0.00 |
| Group Differences in Modulation | From Thal to Thal | 1.00 | 0.66 |
| Group Differences in Modulation | From Thal to IFC | 1.00 | 0.64 |
| Group Differences in Modulation | From SC to CD | 0.00 | 0.00 |
| Group Differences in Modulation | From SC to Thal | 0.00 | 0.00 |
| Group Differences in Modulation | From SC to SC | 0.51 | -0.20 |
| Group Differences in Modulation | From IFC to SEF | 0.00 | 0.00 |
| Group Differences in Modulation | From IFC to CD | 0.00 | 0.00 |
| Group Differences in Modulation | From IFC to Thal | 0.00 | 0.00 |
| Group Differences in Modulation | From IFC to IFC | 0.52 | -0.21 |

Supplementary Figures

Figure S1

Figure S2
